# The H/ACA complex disrupts triplex in hTR precursor to permit processing by RRP6 and PARN

**DOI:** 10.1038/s41467-018-07822-6

**Published:** 2018-12-21

**Authors:** Chi-Kang Tseng, Hui-Fang Wang, Morgan R. Schroeder, Peter Baumann

**Affiliations:** 1 Howard Hughes Medical Institute, 1000 E 50th Street, Kansas City, 64110 MO United States; 2 Stowers Institute for Medical Research, 1000 E. 50th Street, Kansas City, MO 64110 USA; 3 Department of Molecular and Integrative Physiology, University of Kansas Medical Center, Kansas City, KS 66160 USA; 4 Institute of Developmental Biology and Neurobiology, Johannes Gutenberg University, 55099 Mainz, Germany; 5 Institute of Molecular Biology, 55128 Mainz, Germany; 6 Present Address: Institute of Developmental Biology and Neurobiology, Johannes Gutenberg University, 55099 Mainz, Germany; 7 Present Address: ArcherDX, 2477 55th St, Boulder, CO 80301 USA

## Abstract

Human telomerase RNA (hTR) is transcribed as a precursor that is then posttranscriptionally modified and processed. A fraction of the transcripts is oligoadenylated by TRAMP and either processed into the mature hTR or degraded by the exosome. Here, we characterize the processing of 3′ extended forms of varying length by PARN and RRP6. We show that tertiary RNA interactions unique to the longer transcripts favor RNA degradation, whereas H/ACA RNP assembly stimulates productive processing. Interestingly, the H/ACA complex actively promotes processing in addition to protecting the mature 3′ end. Processing occurs in two steps with longer forms first being trimmed by RRP6 and shorter forms then being processed by PARN. These results reveal how RNA structure and RNP assembly affect the kinetics of processing and degradation and ultimately determine the amount of functional telomerase produced in cells.

## Abstract (web summary)

Telomerase RNA (hTR) is transcribed as a 3′-extended precursor. Here the authors examine the processing of hTR precursors of various lengths and show that processing occurs in distinct steps involving different nucleases PARN and RRP6.

## Introduction

The catalytic protein subunit telomerase reverse transcriptase (TERT) and the RNA component telomerase RNA (TR) form the core of the telomerase enzyme. TERT reiteratively copies the template region of TR onto the chromosome ends to replenish terminal DNA repeats lost during DNA replication^1^. In addition to providing the template for telomerase repeats, TR acts as a scaffold for the assembly of protein components that are part of the holoenzyme.

At the gross level, human telomerase RNA (hTR) can be divided into two domains. The 5′ domain folds into an evolutionarily conserved pseudoknot that harbors the template region and a triple helix structure that contributes to telomerase activity^2^. The 3′ half of hTR closely resembles a box H/ACA domain^3^. This structure is composed of two RNA hairpins connected by a single-stranded hinge, referred to as H box (ANANNA). The second hairpin is followed by a 3′ single-stranded tail containing the sequence motif ACA^4^. Like other H/ACA RNAs, the H/ACA domain of hTR is associated with the four H/ACA box proteins dyskerin, NHP2, NOP10, and GAR1^3^. Mutations in the H/ACA domain of hTR have been identified in patients with dyskeratosis congenita, aplastic anemia, and idiopathic pulmonary fibrosis^5^. These have been shown to cause defects in processing and localization of RNA, resulting in accelerated telomere shortening^6,7^. Similarly, mutations in the protein components that interact with this domain also lead to a reduction in the steady-state level of hTR and are associated with the same spectrum of degenerative syndromes^8^.

In vertebrates, H/ACA snoRNAs are typically encoded within introns, and mature via splicing, intron debranching, and exonucleolytic processing^4,9^. In contrast, hTR is transcribed as an independent gene by RNA polymerase II and is subsequently processed into the 451-nt mature form. While the promoter of hTR is well-characterized^10^, the mechanism of transcription termination and processing remain poorly understood. Several independent studies have demonstrated the existence of hTR transcripts extending beyond the mature end at position 451^7,11–15^. Some 3′-extended forms are only a few nucleotides (nts) longer than the mature hTR, whereas others exceed 1500 nts in length. Their accumulation in cells depleted of the cap-binding complex (CBC), the nuclear exosome targeting complex (NEXT), and the exosome suggests that the long forms are predominantly degraded by this pathway^11,12^. Whether a fraction of the long transcripts is converted into shorter intermediates or whether short primary transcripts are also generated has remained unclear.

A key insight into hTR processing came from genetic studies linking poly(A)-specific ribonuclease (PARN) to telomere dysfunction in patients with dyskeratosis congenita^13,16–18^. Cells from patients with mutations in PARN have short telomeres and reduced levels of hTR^13,16–20^. Oligo-adenylated hTR that extends beyond the mature 3′ end by a few nucleotides was found to be increased in these cells^12,19^. Oligo-adenylation is predominantly mediated by PAPD5 (TRF4-2), a non-canonical poly-A polymerase that is a component of the TRAMP complex^21^. Oligo-adenylation of some RNAs by TRAMP stimulates degradation by the exosome^22,23^. This has led to a model in which PARN antagonizes degradation by removing oligo (A) tails from hTR. Whether PARN or another nuclease trims hTR to position 451 has remained controversial. To address this issue and to elucidate which factors determine whether an hTR precursor RNA is acted upon by PARN or the exosome, we conducted a series of experiments to examine the processing of RNAs of varying lengths. These studies establish direct roles for PARN and RRP6 in hTR processing, reveal a novel function for the H/ACA complex in promoting processing, and identify a role for a triple helix structure in competing with ribonucleoprotein complex (RNP) assembly.

## Results

### Effect of hTR sequence on PARN-mediated processing

**Fig. 1:**
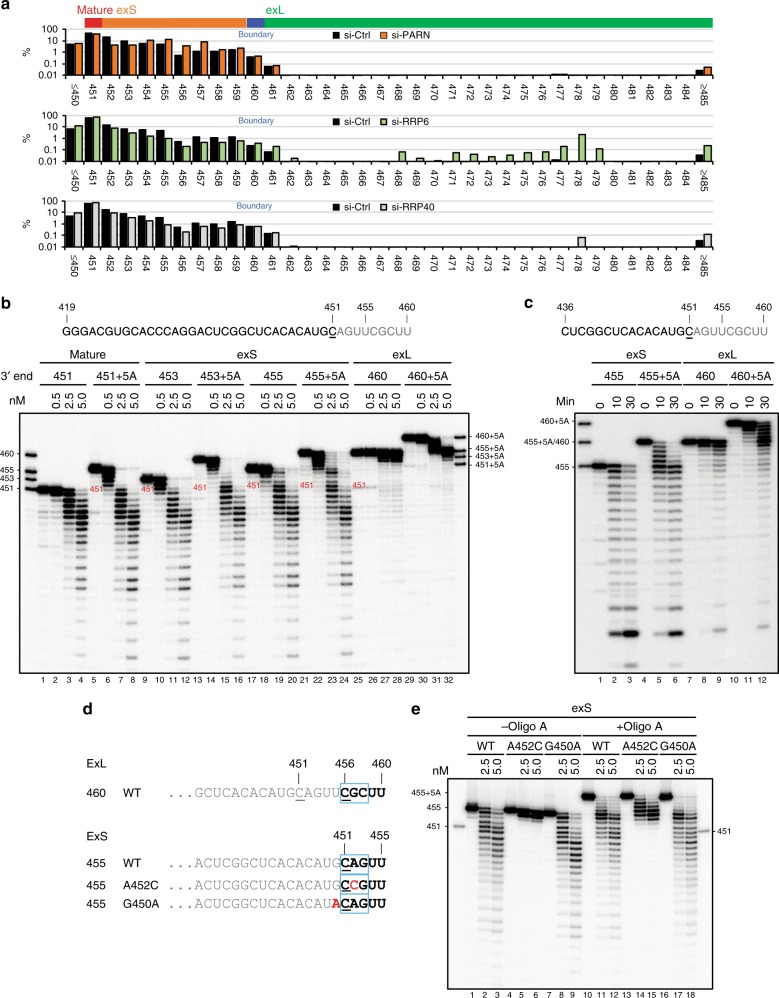
Effect of hTR sequence composition on PARN-mediated processing. **a** Bar graph showing distribution of hTR 3′ end positions with respect to gene sequence excluding non-templated adenosines present on a subset of the transcripts. Total RNA was prepared from HeLa cells treated with siRNAs targeting PARN, RRP6, and RRP40, respectively. Non-targeting siGENOME pool was used as control. The 3′ ends of hTR were mapped using RNA ligase-mediated 3′ RACE. Number of reads analyzed: si-Ctrl (PARN), 1,000,754; si-PARN, 761,630; si-Ctrl (RRP6), 896,607; si-RRP6, 982,658; si-Ctrl (RRP40), 790,476; si-RRP40, 730,951. **b** 5′-^32^P-labeled hTR oligos (nucleotide 419 to indicated 3′ end with or without oligo (A) tails) were incubated without or with 0.5, 2.5, or 5.0 nM of recombinant PARN at 37 ^o^C for 30 min. The reaction products were resolved on a 20% polyacrylamide gel under denaturing conditions. **c** 5′-^32^P-labeled variants of hTR oligo (nucleotide 436 to 455 or 460 with or without oligo A tails) were incubated with 100 pM of recombinant PARN for the indicated times. **d** Schematic of hTR oligos used in **e**. **e** 5′-^32^P-labeled variants of hTR oligos were incubated without or with 0.5, 2.5, or 5.0 nM of recombinant PARN at 37 °C for 30 min. The reaction products were resolved on a 20% polyacrylamide gel under denaturing conditions

To assess the fate of 3′-extended forms of hTR, we depleted PARN, the exosome-associated nuclease RRP6, and the core exosomal component RRP40, respectively, from cultured cells (Supplementary Figure 1a) and analyzed the distribution of hTR 3′ ends by RNA ligase-mediated rapid amplification of cDNA ends (RLM-RACE) coupled with Illumina sequencing. The analysis revealed distinct categories of 3′ extended RNAs. Knockdown of PARN resulted in an increase of RNA terminating at positions 454–457, coincident with a reduction in the fraction of shorter transcripts ending at positions 452–453 (Fig. 1a, top panel and Supplementary Figure 1b, top panel). Knockdown of RRP40 and RRP6 resulted in an increase in the fraction of longer hTR transcripts with 3′ termini mapping beyond position 460, concomitant with a reduction in the fraction of short hTR transcripts that terminate at positions 452–459 (Fig. 1a, middle and bottom panels and Supplementary Figure 1b, middle and bottom panel). The accumulation of 460+ isoforms was more pronounced in the RRP6 knockdown than the RRP40 knockdown. Consistent with prior reports, knockdown of PARN caused a reduction in mature hTR, whereas knockdown of RRP6 or RRP40 resulted in an increase in the mature form^12,13,17–20^. As these length-specific phenotypes suggested the existence of functionally distinct RNA isoforms, we will henceforth refer to hTR molecules that terminate between positions 452 and 460 as the 3′-extended short (exS) form and those molecules that terminate beyond position 460 as the 3′-extended long (exL) form. Whereas RRP6 appears to primarily act on the exL forms, PARN seems to function either by antagonizing degradation of oligo-adenylated exS or by actively processing exS into the mature form.

To gain further insights into the mechanisms of PARN action, we generated a series of synthetic RNA oligonucleotide substrates. PARN has a strong preference for degrading homopolymers of adenosine over other nucleotides^24–26^. However, it was not known whether following removal of an oligo-A tail, the enzyme would also remove genome-encoded sequences from hTR to generate the mature 3′ end. Oligo (A) sequences were efficiently trimmed from all RNA substrates (Fig. 1b). Further degradation was observed for the mature form (ending at position 451) and exS forms (ending at position 453 or 455). In contrast, an exL form (ending at 460) was resistant to PARN even at higher concentration (lanes 25–32). PARN also degraded mature and exS substrates not carrying an oligo (A) tail but did not act on the exL RNA. A time course revealed that adenylated versions of mature and exS were more rapidly deadenylated by PARN than exL (Supplementary Figure 2a). The inefficient processing of exL RNA is not simply a consequence of exL being a longer RNA, as exL was still resistant to PARN after removing 17 nts from the 5′ end of the substrate (Fig. 1c). These results show that PARN preferentially acts on exS in vitro, consistent with the in vivo observation that exS forms accumulate following PARN depletion.

The observed substrate specificity pointed toward sequence-dependent attenuation of PARN. The enzyme has been shown to exhibit a tenfold preference for oligo (A) over oligo (U), with oligo (C) and oligo (G) being even poorer substrates^24–26^. Surprisingly though, the exS (455) and the exL (460) forms both terminate in two uridines. The sequences diverge only further upstream: (456-CGC-458) in exL and (451-CAG-453) in exS. To determine whether GC-rich sequence inhibits PARN, we introduced A452C, A452G, and G450A mutations into exS substrates (Fig. 1d and Supplementary Figure 2b and 2c). A452C generates the sequence CCG and A452G generates CGG. In particular, A452C strongly decreased the efficiency of deadenylation and degradation by PARN, with A452G causing modest attenuation of PARN (Fig. 1e, and Supplementary Figure 2c). By contrast, deadenylation and degradation of G450A was slightly more efficient than wild type (Fig. 1e, compare lanes 3 and 9). These results indicate that PARN activity is affected by sequence context at a distance of several nucleotides.

### The H/ACA RNP attenuates processing by PARN at position 451

**Fig. 2:**
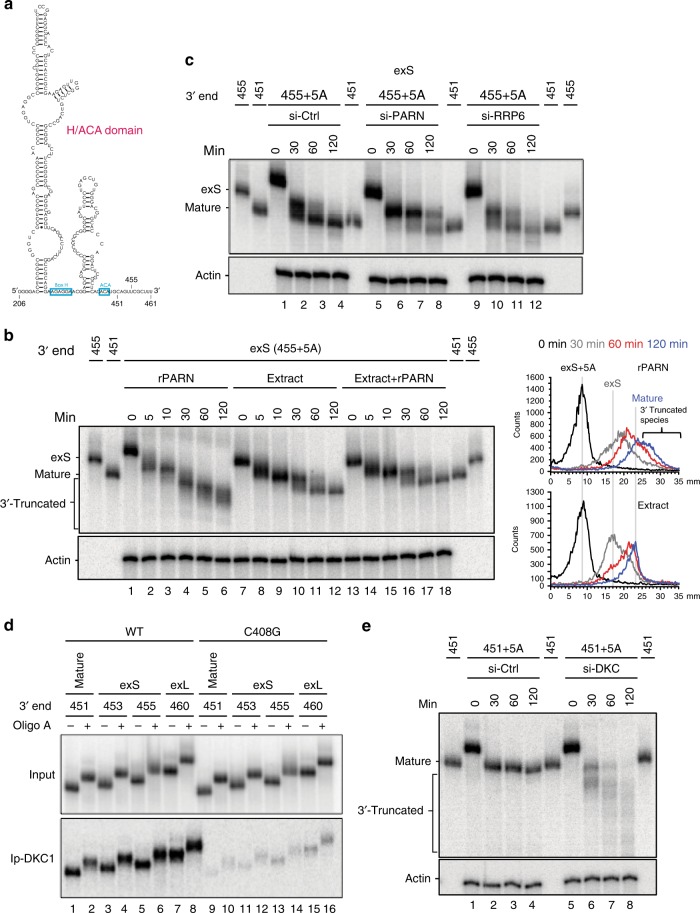
PARN processing is attenuated by the H/ACA RNP. **a** Schematic of the hTR fragment used in the in vitro assay. **b** ^32^P-labeled hTR fragments (nucleotides 206 to 455 with oligo A tails) were incubated with 5.0 nM of recombinant PARN alone or in HeLa cell extracts without or with 5.0 nM of recombinant PARN at 37 °C for the indicated times. RNA was purified and resolved by a 6% polyacrylamide/8 M urea gel. **c** The ^32^P-labeled in vitro transcribed hTR fragments (nucleotides 206 to 455 with oligo A tails) was incubated in cell extracts (20 μg) prepared from siRNA-treated cells as indicated. The reaction was performed at 37 °C for the indicated times. RNA was purified and resolved by a 6% polyacrylamide/8 M urea gel. **d** Wild type or C408G mutant hTR fragments (nucleotides 206 to 451, 453, 455, and 460 with or without oligo A tails) were in vitro transcribed in the presence of α-^32^P-UTP and incubated in HeLa cell extracts on ice for 10 min. The reaction mixture was immunoprecipitated with antibodies against dyskerin. RNA was purified and resolved by a 6% polyacrylamide/8 M urea gel. **e** The ^32^P-labeled in vitro transcribed hTR fragments (nucleotides 206 to 451 with oligo A tails) were incubated in cell extracts (20 μg) prepared from siRNA-treated cells as indicated. The reaction was performed at 37 °C for the indicated times. RNA was purified and resolved by a 6% polyacrylamide/8 M urea gel

Although in vitro processing assays revealed pronounced effects of sequence on PARN processivity, little attenuation was observed at the position of the mature 3′ end (Fig. 1b). Clearly, sequence context alone is not sufficient for PARN to generate the 451-nt mature RNA. To examine whether folding of the RNA into the two-hairpin structure of an H/ACA box RNA is needed, we in vitro synthesized the H/ACA domain of hTR (starting at nucleotide 206) as mature, exS and exL forms (Fig. 2a). Incubation of recombinant PARN with H/ACA domain exS plus oligo (A) tail resulted in products shorter than the mature form, demonstrating that the hairpin structure alone is insufficient to attenuate PARN at position 451 (Fig. 2b, lanes 1–6). In contrast, the mature form was produced during incubation with whole cell extract (lanes 7–12), even when the extract was supplemented with excess recombinant PARN (lanes 13–18). To confirm that the 3′ processing of the hTR precursor is mediated by PARN, extracts were prepared from cells subjected to PARN knockdown and control siRNA (Supplementary Figure 3a, lane 2). The 3′ processing of the precursor into mature hTR was substantially reduced in extracts from PARN knockdown cells (Fig. 2c, lanes 5–8 and Supplementary Figure 3b and 3c). Although deadenylation still neared completion in less than 30 min, further processing was inefficient, and after 120 min, less than 25% had been converted into the mature form. These results further support a role for PARN in converting exS into the mature form.

As binding of the H/ACA complex is critical for hTR stability in vivo, we next asked whether this complex was responsible for attenuating PARN at position 451 in vitro. Following incubation of mature, exS and exL in extracts on ice, all forms of wild-type but not C408G mutant hTR were recovered by immunoprecipitation with anti-DKC1 (Fig. 2d). Consistent with previous results, siRNA-mediated knockdown of dyskerin reduced the steady-state level of hTR in cells by tenfold (Supplementary Figure 4a and 4b). Incubation of oligo-adenylated hTR (nucleotides 206–451+5A) in control extracts at 37 °C confirmed that this form is stable over 2 h following rapid deadenylation (Fig. 2e, lanes 1–4). In contrast, this RNA was rapidly degraded in the dyskerin knockdown extracts (Fig. 2e, lanes 5–8 and Supplementary Figure 6), indicating that the in vitro assay recapitulates the protective role of the H/ACA RNP bound to hTR in cells.

### RRP6 and dyskerin are required for the processing of exL

**Fig. 3:**
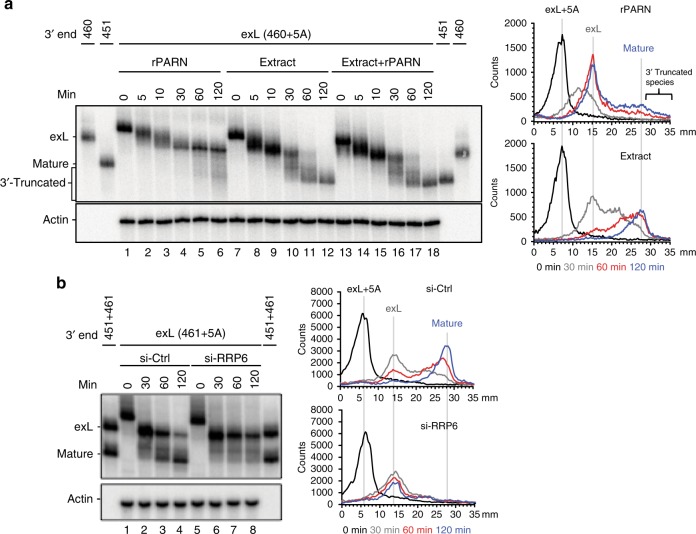
The role of RRP6 in the processing of exL. **a** The ^32^P-labeled in vitro transcribed hTR fragments (nucleotides 206 to 461 with oligo A tails) were incubated with 5.0 nM of recombinant PARN or in HeLa cell extracts (20 μg) without or with 5.0 nM of recombinant PARN at 37 °C for the indicated times. RNA was purified and resolved on a 6% polyacrylamide/8 M urea gel. **b** The ^32^P-labeled in vitro transcribed hTR fragments (nucleotides 206 to 461 with oligo A tails) were incubated in extracts prepared as indicated in Supplementary Figure 1b. The reaction was performed at 37 ^o^C for the indicated times. RNA was purified and resolved on a 6% polyacrylamide/8 M urea gel

Recombinant PARN alone failed to degrade exL oligonucleotides (Fig. 1b). Similarly, incubation of the exL form of the H/ACA domain (460+5A) resulted in deadenylation but lack of further processing, although a smear indicating some degradation at the later time points (Fig. 3a, lanes 1–6). In contrast, incubation with whole cell extract produced the mature form (Fig. 3a, lanes 7–18), indicating that 3′ processing of exL requires activities in addition to PARN. This idea was also supported by the observations that PARN knockdown resulted in an increase in the fraction of hTR exS with minimal effect on exL (Fig. 1a, top panel). In contrast, knockdown of RRP6 resulted in a pronounced increase in exL, indicating a role for this nuclease in the processing of longer forms (Fig. 1a, middle panel).

**Fig. 4:**
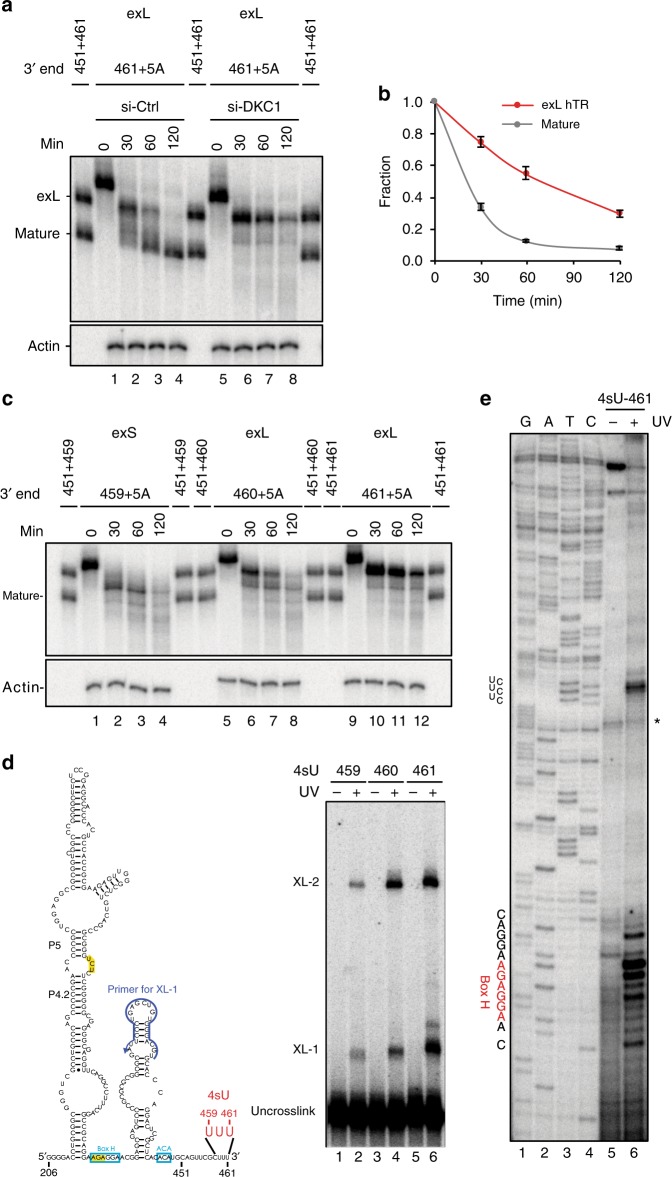
A triplet helix structure forms in exL. **a** The ^32^P-labeled in vitro transcribed hTR fragments were incubated in extracts prepared from 293T cells subjected to dyskerin knockdown and control siRNA for the indicated times. RNA was purified and resolved by a 6% polyacrylamide/8 M urea gel. **b** The ^32^P-labeled in vitro transcribed hTR fragments were incubated in dyskerin knockdown extracts for the indicated times. RNA was purified and resolved on a 6% polyacrylamide/8 M urea gel. The hTR signal was quantified using a phosphoimager. The ratio of each RNA species was normalized to both time 0 and the loading control, Actin. Mean values were calculated from three biological replicate experiments as shown in Supplementary Figure 6. Bars represent the standard error. For the mature form, 50% is degraded after 22.4 min, for exL after 69.9 min. **c** The ^32^P-labeled in vitro transcribed hTR fragments were incubated in dyskerin knockdown extracts for the indicated times. RNA was purified and resolved on a 6% polyacrylamide/8 M urea gel. **d** Schematic showing an hTR fragment with a single nucleotide replaced with 4-thiouridine (4sU) for positions 459, 460, and 461 used for the in vitro UV crosslinking assay. Nucleotides modified with 4sU are shown in red. The primers used for primer extension are shown in blue. RNA crosslinks were induced by irradiation with UV light. The crosslinked products were resolved on a 6% polyacrylamide/8 M urea gel. **e** Specific signals corresponding to XL-1 were gel-purified and mapped by primer extension. Asterisks mark non-specific RT pause sites

To examine the requirement of RRP6 in processing or degradation of exL, we prepared extracts from cells that had been subjected to RRP6 siRNA (Supplementary Figure 3a, lane 3). Oligo-adenylated exS was still processed into mature hTR in the knockdown extract (Fig. 2c, lanes 9–12 and Supplementary Figure 3b and 3c). In contrast, oligo-adenylated exL was deadenylated but not processed into mature hTR following knockdown of RRP6 (Fig. 3b and Supplementary Figure 5). Together with the in vivo 3′ end sequence analysis, these results argue for exL first being processed by RRP6 into exS, which then becomes a substrate for PARN to generate the mature form. This raises the question of what determines whether exL is degraded or processed into exS? In vivo, cotranscriptional assembly of the H/ACA core complex on hTR is thought to be critical for protecting hTR from degradation^27–29^. To examine the effect of H/ACA RNP assembly on exL processing, oligo-adenylated exL (nucleotides 206–461+5A) was incubated in the siRNA-control and dyskerin knockdown extracts, respectively (Fig. 4a). Whereas deadenylation was unaffected by the presence or absence of dyskerin (Fig. 4a, compare lanes 2 and 6), the processing into the mature form was abolished in the dyskerin knockdown extracts (Fig. 4a, lanes 5–8). Although the exL form was partially degraded in the absence of dyskerin, it is important to note that the exL form is threefold more stable than mature hTR in the absence of dyskerin (Fig. 4b and Supplementary Figure 6). The deficiency in 3′ processing following dyskerin knockdown was not caused by a reduction in PARN or RRP6 (Supplementary Figure 4a). These observations demonstrate that the H/ACA core complex is not only essential for the protection of hTR from degradation but is also required for processing of exL into exS. This indicates that an RNA structure or an RNA binding protein selectively stabilizes the exL forms when not bound by the dyskerin complex.

### Tertiary RNA interactions stabilize exL

To dissect the basis for the increased stability of exL in DKC1 knockdown extracts, we examined the stability of oligo-adenylated RNAs ending at positions 459, 460, and 461, respectively (Fig. 4c). All three substrates were deadenylated, but the additional two nucleotides at the 3′ terminus of 461 resulted in notable stabilization relative to the RNA ending at position 459 (compare lanes 4 and 12). Increased resistance to degradation by 3′–5′ exonucleases could be mediated by RNA secondary structure involving the 3′ terminal sequence. To test this idea, we utilized an RNA crosslinking method in which the modified ribonucleotide 4-thiouridine (4sU) was introduced into the exL (461) form at positions 459, 460, and 461, respectively (Fig. 4d). Under exposure to UV light, 4sU reacts with nearby RNA bases, and crosslinked products can be separated from uncrosslinked RNA by denaturing gel electrophoresis. Two main conjugate bands (XL-1 and XL-2) appeared specifically after UV-irradiation (Fig. 4d). The crosslinked products were most abundant when 4sU was introduced at position 461 (lane 6). To identify the interaction site, crosslinked products were gel-purified and mapped by primer extension. We were unable to map the positions crosslinked in XL-2, which may represent interactions between two RNA molecules. Remarkably though, the XL-1 band revealed intramolecular crosslinks at two specific locations, the H box and the UCU sequence between the P4.2 and P5 stems (Fig. 4e, lane 6). As the UCU sequence cannot base pair with the UUU sequence at the 3′ end of hTR (461), the crosslinks are indicative of triple base interactions such as U–A●U found in triple helix structures (where ● denotes the Hoogsteen face and – denotes the Watson–Crick face).

### Role of the tertiary interactions in hTR biogenesis

**Fig. 5:**
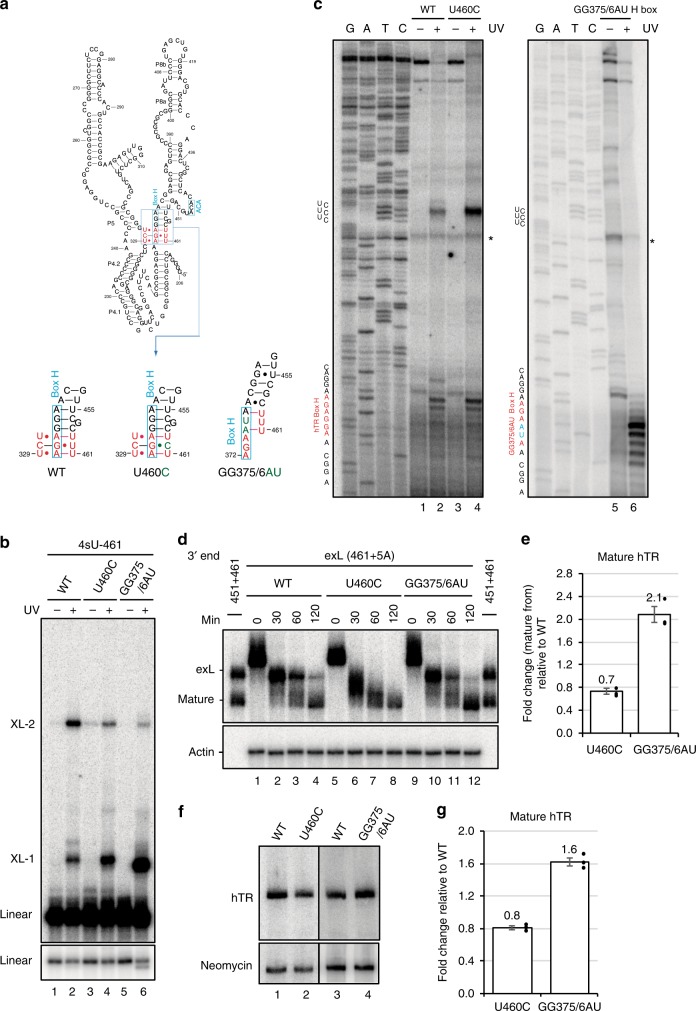
The importance of the triple helix structure in biogenesis of hTR. **a** Schematic of the proposed triple helix structure formed in exL. Introduced nucleotide replacements in the H box motif are depicted. **b** Wild type, U460C, and GG375/6AU hTR transcripts with a single radioactive phosphate followed by a single photoactivatable 4sU at position 461 were irradiated with UV light, followed by separation on a 6% polyacrylamide/8 M urea gel. **c** Specific signals corresponding to XL-1 were gel-purified and mapped by primer extension. Asterisks mark non-specific RT pause sites. **d** and **e** In vitro hTR processing assays were performed with the ^32^P-labeled hTR fragments as indicated in **a**. RNA was purified and resolved on a 6% polyacrylamide/8 M urea gel. The bar graph depicts mean fold change for mature form relative to wild-type samples and normalized to Actin. Mean values were calculated from in vitro hTR processing assays of three biological replicates. Bars represent the standard error. Significance of change in mature form between samples was calculated with a two-sided Student’s *t*-test; *p* values: 0.002462 (U460C); 0.001484 (GG375/6AU). Dots represent data points from individual experiments. **f** and **g** VA13 cells were transfected with vectors containing wild type, U460C, or GG375/6AU mutant. Total RNA was prepared and subjected to northern blot. A probe against neomycin served as control for transfection efficiency and loading. The bar graph illustrates the mean fold change for hTR levels relative to wild-type samples and normalized to neomycin. Mean values were calculated from northern blot experiments of three biological replicates. Bars represent the standard error. Significance of change in mature form relative to wild-type samples was calculated with a two-sided Student’s *t*-test; *p* values: 0.000989 (U460C); 0.000239 (GG375/6AU). Dots represent data points from individual experiments

Engaging the 3′ end and the H box in tertiary RNA interactions would not only impair 3′ exonucleolytic degradation, but also compete with H/ACA RNP assembly on the H-box. To assess the effects of stabilizing or destabilizing this new structure, two mutants were created. The U460C mutant strengthens a triple base interaction by changing C–G●U (C318, G373, and U460) to C–G●C, providing an additional hydrogen bond (Fig. 5a and Supplementary Figure 7)^30^. To disrupt the tertiary base interactions, GG375/6 was replaced with AU (Fig. 5a). This particular mutant was chosen as it converts the hTR H box into the equivalent motif of snoRNA U92, thus maintaining a functional dyskerin-binding site while disrupting the potential for triple helix formation. Where U460C decreased dyskerin binding, the GG375/6AU mutations resulted in increased dyskerin binding (Supplementary Figure 8). UV-crosslinking with 4sU at position 461 produced an enhanced XL-1 signal for U460C as expected for a stabilized structure (Fig. 5b). Mapping of the crosslink sites corroborated enhanced interaction between the exL 3′ end and the UCU sequence starting at position 327 (Fig. 5c). Although the GG375/6AU mutant showed a strong crosslink near XL-1 (Fig. 5b), mapping revealed simple base pairing with the H box and no detectable tertiary interaction with the 327-UCU sequence (Fig. 5c).

Subjecting the U460C mutant to the in vitro processing assay resulted in a 30% increase in degradation compared to wild type (Fig. 5d, e). Similarly, introduction of hTR containing the U460C mutation into VA13 cells resulted in a 20% decrease in the steady-state level of the mature hTR in vivo (Fig. 5f, g). This is the first mutation downstream of position 451 shown to affect the steady-state level of the RNA, suggesting that mutations downstream of position 451 could also be associated with premature telomere shortening in humans. The GG375/6AU mutation had the opposite effect on hTR processing, causing an approximately twofold increase in the mature form in vitro (Fig. 5d, lanes 9–12 and Fig. 5e) and an ~1.6-fold increase in vivo (Fig. 5f, g). In aggregate, these results reveal that the exL form of hTR can adopt a triple helix structure. Conversion of the exL form into exS requires binding of the H/ACA complex to shift the balance from degradation to maturation.

## Discussion

**Fig. 6:**
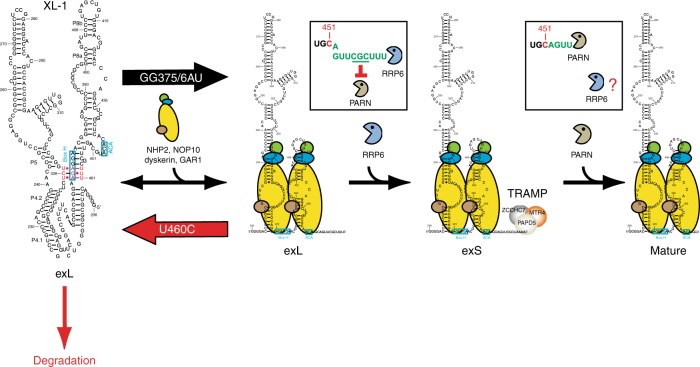
Model. Schematic illustrating the multiple roles of the H/ACA complex and the different functions for RRP6, the exosome, and PARN in human telomerase RNP biogenesis

The correct processing of TR and the assembly of the RNP are critically important for enzymatic activity and telomere length homeostasis. Here, we have shown that hTR 3′ end processing involves two distinct precursors that are trimmed by different nucleases, a process that is in kinetic competition with degradation of the RNA. Our studies revealed that the H/ACA proteins have multiple distinct roles during biogenesis. Firstly, they compete with a triple helix structure formation; secondly, their binding results in conformational rearrangements in exL that promotes processing into exS by RRP6; and thirdly, the H/ACA complex also functions in the conversion of exS into the mature form by attenuating PARN at position 451 (Fig. 6).

The observed increase in oligo-adenylated mature hTR in cells with reduced PARN activity supports a model in which oligo-adenylation of hTR by the TRAMP complex targets it for degradation by the exosome. Previous studies had concluded that PARN rescues some hTR from the exosome by removing A tails^12,20^. We now provide evidence for an additional function of PARN in the processing of the short precursor exS. We show that PARN is indeed able to trim genome-encoded sequence from the 3′ end of exS, albeit with lower processivity than the degradation of oligo (A) tails. Nevertheless, in the absence of PARN, processing of exS into mature hTR is inefficient (Fig. 2c), arguing for a direct role for PARN in this processing step in cells. A recent study implicated the nuclease TOE1 in functioning with PARN in the processing of some RNA substrates including snoRNAs, scaRNAs, and hTR^31^. Whether TOE1 mutations affect telomere length and maintenance remains to be investigated.

Another area in need of further investigation is the mechanism and sites of transcription termination. Intriguingly, examination of processing intermediates that accumulate in cells after siRNA knockdowns revealed that 3′ end processing proceeds in at least two distinct steps (Fig. 6). First, RRP6 acts on exL to convert it into exS. The observation that exL accumulates in RRP6 siRNA-treated cells to a much greater extent than following RRP40 knockdown is noteworthy, especially since RRP40 knockdown results in a pronounced reduction of RRP6 as well as RRP40^12^. While we cannot rule out the possibility that the RRP6 knockdown is more efficient than the RRP40 siRNA, the observation is also consistent with an exosome-independent function of RRP6 in the processing of exL. A role for RRP6 that is independent of the core exosome is not without precedent. In vivo RNA–protein crosslinking (CRAC) analysis of yeast exosome components revealed substrates only enriched in the Rrp6 data set^32^. For example, truncated 18s rRNA transcripts appear to be degraded in a process that involves Trf4 and Rrp6, but not the core exosome^32^. In the context of TR, it is tempting to speculate that exL processing may be carried out by RRP6 alone, whereas degradation involves the entire exosome. Consistent with this idea, reverse transcription quantitative polymerase chain reaction (RT-qPCR) of hTR implicated the core exosome (RRP40) and its two associated exonucleases, RRP6 and RRP44/DIS3, in the turnover of hTR extending by at least 50 nts beyond the mature 3′ end^11,12^. In one study, knockdown of RRP40 stabilized 3′ extended hTR sixfold more than knockdown of RRP6. The implication of the NEXT complex in the turnover of longer forms of hTR^12^ supports the idea that degradation occurs via NEXT targeting the exosome to hTR, whereas processing may involve RRP6 alone or in complex with other factors.

It has become clear that RNA triple helix structures affect many aspects of RNA biology and may be far more common than previously thought^33^. They have been found in diverse functional contexts. For instance, the catalytic activity of telomerase depends on a triple helix in the pseudoknot domain of hTR^2,34^. Furthermore, a triple helix in the SAM-II riboswitch creates a binding pocket for *S*-adenosylmethionine^35^, and triple helix structures have also been implicated in protecting 3′ ends of RNAs from exonucleolytic degradation. In the case of the Kaposi’s sarcoma-associated herpes virus polyadenylated nuclear RNA, a protective triple helix involves the poly(A) tail^36^. In MALAT1 RNA, a triple helix structure involving the non-polyadenylated 3′ end increases RNA stability and stimulates translation^37,38^. The tertiary RNA interactions involving the 3′ end of hTR exL, the H box, and an internal loop sequence does not fit into any of these functional paradigms. Although the structure inhibits rapid nuclear RNA decay as shown for the analogous structure in MALAT1, its presence in hTR is ultimately in direct competition with canonical processing. Functionally, it appears that the structure transiently protects exL from rapid degradation and creates a window of opportunity for the H/ACA complex to bind and remove the triple helix in the process. Consistent with dyskerin binding resolving the triple helix structure, exL has a notably longer half-life in extracts from dyskerin-depleted cells than exS or the mature form (Fig. 4b). The discovery of METTL16 as a triple-helix RNA binding protein of MALAT1^39^ raises the possibility that dyskerin or another factor is preferentially recruited to exL in the triple helix conformation. Depending on the context, this may advance processing or target the RNA for degradation.

Independent of the formation of the triple helix structure, PARN is inhibited at the boundary of exL and exS by sequence context. Attenuation of both nucleases by sequence and structure allows time for conformational rearrangements and RNP assembly, thereby coupling successful H/ACA RNP formation to the final processing steps. This creates an effective quality-control pathway for hTR biogenesis but makes the process of generating sufficient telomerase to ensure long-term telomere length homeostasis sensitive to defects in a large number of RNA processing factors. Ongoing association studies will undoubtedly uncover additional loci associated with telomeropathies.

## Methods

### In vitro deadenylation

Trace amounts of 5′-^32^P-labeled (3000 cpm µl^−1^) and 250 nM unlabeled hTR RNA oligo were incubated with the indicated concentrations of recombinant PARN (Origene, cat. # TP307220) in a buffer containing 20 mM Tris-HCl (pH 7.5), 50 mM KCl, 2.5 mM MgCl_2_, 50 µg ml^−1^ BSA (NEB), and 40 U RNasin plus RNase inhibitor (Promega). A 10-µl reaction mixture was incubated at 37 °C for 30 min, and the reaction was stopped by the addition of 2× RNA loading dye containing 99% formamide, 0.025% xylene cyanol, 0.025% bromophenol blue, and 0.1 mM EDTA. The samples (5 µl) were separated on 20% denaturing acrylamide sequencing gels containing 7 M urea at 80 W for 3 h for long hTR RNA oligos and 1.5 h for short hTR oligos. RNA oligos used for in vitro deadenylation assays are listed in Supplementary Tables 1 and  2.

### Preparation of hTR RNA substrates and in vitro hTR processing assay

In vitro transcription reactions were carried out in 1× transcription buffer (Promega), 0.5 mM each for ATP, CTP, and GTP, 0.1 mM UTP, 0.66 µM α-^32^P-UTP (3000 Ci mmol^−1^, 10 mCi ml^−1^, PerkinElmer), 1 µg DNA template, 40 U RNasin plus RNase inhibitor (Promega), and 20 units T7 RNA polymerase (Promega). Reaction mixtures (10 µl) were incubated at 37 °C for 2 h followed by the addition of an equal volume of formamide dye. Full-length RNA products were purified on 8% polyacrylamide/8 M urea gels. Primers used to generate DNA templates are listed in Supplementary Table 3. For preparation of the loading control actin mRNA, a similar reaction was carried out except that pSP6Act6-88 was used as DNA template and 18.9 units of Sp6 RNA polymerase (Promega) instead of T7 RNA polymerase.

In vitro hTR processing reactions (10 µl) were carried out at 37 °C in a buffer containing 20 mM Tris-HCl (pH 7.5), 50 mM KCl, 2.5 mM MgCl_2_, 50 µg ml^−1^ BSA (NEB), 40 U RNasin plus RNase inhibitor (Promega), 2 nM ^32^P-labeled hTR RNA, and either indicated amounts of recombinant PARN or 20 µg of whole cell extract, or both. Reactions were stopped by the addition of 10 µl stop buffer (10 mg ml^−1^ proteinase K in 0.5% SDS, 40 mM EDTA, 20 mM Tris-HCl, pH 7.5, and 1000 cpm µl^−1 32^P-labeled actin mRNA) and incubation at 37 °C for 20 min followed by extraction with phenol/chloroform pre-equilibrated with 50 mM NaOAc (pH 5.0), and ethanol precipitation. RNA was dissolved in 70% formamide dye and analyzed on 6% polyacrylamide (19:1) gels containing 8 M urea.

### Constructs and site-directed mutagenesis

A fragment of DNA containing 1.5 kb upstream and 2 kb downstream of the major transcription start site of hTR was cloned into pACGFP1-1 using XhoI and BamH1 sites; this plasmid was named pMG80. Mutations were introduced using QuikChange II XL Site-Directed Mutagenesis Kit (Agilent). Oligonucleotides used for site-directed mutagenesis are listed in Supplementary Table 4.

### Cell culture, transfection, and sample preparation

WI-38 VA-13 subline 2RA cells (ATCC^®^ CCL-75.1^™^) were cultured in E-MEM medium (ATCC, 30-2003) supplemented with 10% fetal bovine serum (Sigma-Aldrich) at 37 °C, 5% CO_2_. Cells were transfected with 12 µg of plasmids using Fugene HD (Promega) for 72 h. Plasmids used for transfection are listed in Supplementary Table 5. HeLa cells were cultured in D-MEM (Life Technologies, 11995-065) containing 10% fetal bovine serum (Sigma-Aldrich) at 37 °C, 5% CO_2_. 293 T cells (ATCC^®^ CRL-3216^™^) were cultured in DMEM medium (Life Technologies, 11995-065) containing 10% fetal bovine serum (Sigma-Aldrich) at 37 °C, 5% CO_2_. HeLa or 293T cells were treated with 20 nM or 40 nM siRNA for 72 h using Dharmafect I (Dharmacon). siRNAs used in this study are listed in Supplementary Table 6.

### Western blot

For western blotting analysis, cells were lysed in a solution containing 2× LDS sample buffer (Life Technologies), 0.78% β-mercaptoethanol (v/v) (J.T. Baker), and 4% SDS. Protein lysate was loaded onto a 4–12% Bis-Tris protein gel (Invitrogen) and transferred to a nitrocellulose blotting membrane (GE Healthcare Life science, 10600012), Low-fat milk (5%) in TBS buffer was used as a blocking reagent. Antibodies used in this study are listed in Supplementary Table 7.

### Northern blot

Cells were collected in Trizol reagent (Ambion, Life Technologies) according to the manufacturer’s instructions, followed by treatment with DNase I (New England Biolabs) at 37 °C for 30 min. Total RNA was extracted once with phenol/chloroform equilibrated with 50 mM NaOAc (pH 5.0), then ethanol precipitated. RNA (10 µg) was separated on a 4% polyacrylamide gel containing 8 M urea at 15 W for 1.5 h. RNA was transferred to a hybond-N1 nylon membrane (GE Healthcare Life science) at 400 mA for 1 h followed by crosslinking in a Stratalinker (Stratagene, 254 nm, 120 mJ). Hybridizations were carried out in Church-Gilbert buffer at 65 °C (for hTR, probes were generated by nick translation of a polymerase chain reaction (PCR) fragment with ^32^P-α-dCTP) and 42 °C (for oligonucleotide probe BLoi2948 against neomycin, labeled with ^32^P-γ-ATP by T4 PNK kinase). Oligonucleotide sequences are listed in Supplementary Table 8.

### RNA ligase-mediated 3′ RACE and deep sequencing

Library preparation was based on a published protocol^12^. Oligonucleotides used for the library preparation are listed in Supplementary Table 9. Ligation reactions contained 1.5 μg total RNA, 1× T4 RNA ligase buffer, 3.7% PEG8000, 7.4% DMSO, 10 U T4 RNA ligase I, 0.7 mM ATP, and 3.7 μM 3′ linker (BLoli5511). The ligation reaction mixture was incubated at 16 °C for 18 h and inactivated at 65 °C for 15 min. About 30 μl of reverse transcription reaction contained ligation reactions, 1× First Strand buffer, 1.6 μM RT primer (BLoli5575), 0.5 μM dNTPs, 5 mM DTT, 40 U RNasin plus RNase inhibitor (Promega), and 300 U Superscript III (Invitrogen) and was incubated at 55 °C for 60 min. A total of 10 U RNase H was directly added into the reverse transcription reaction, followed by incubation at 37 °C for 20 min and then heat-inactivation at 70 °C for 15 min. The first round PCR was performed with 0.5 μM BLoli5575 and BLoli5574 in 50 μl volumes containing 1× Phusion HF buffer, 0.2 mM dNTPs, and 1 U Phusion Hot Start II polymerase (Thermo Scientific). First-round PCR products were purified by PCR purification kit (QIAGEN). For the second-round PCR reactions, 5 μl of 10×-diluted first-round PCR product was used as a template; 0.5 μM BLoli4666 was used as the forward primer for all samples and reverse primers BLoli4668, BLoli4669, BLoli4779, BLoli4780, BLoli4782, and BLoli4783 were used for si-Ctrl (PARN), si-PARN, si-Ctrl (RRP6), si-RRP6, si-Ctrl (RRP40), and si-RRP40, respectively. Amplicons were purified by PCR purification kit (QIAGEN) and the primer dimers were removed using the Pippin Prep System (Sage Science). The samples were quantified by Qubit and Bioanalyzer, multiplexed and split between two lanes of a RapidSeq flow cell for sequencing. The library samples were sequenced on the Illumina HiSeq using RapidSeq-250 bp single-end reads. A 10-nt molecular barcode was used to remove PCR duplicates. After quality filtering, 0.7 to 1.0 million reads were analyzed per sample. To pass the filter, a read required a minimum match of 20 nts to hTR reference sequence and 10 nts to the linker sequence. For each filtered read, the most 3′ hTR reference coordinate between hTR:366–641 was identified by matching 20 nts closest to the 3′ end allowing for two mismatches not including the two most 3′ bases. Nucleotides found between the reference hTR and the linker sequence were considered non-templated nucleotide additions (NTNA). All reads were included in the 3′ end analysis irrespective of the presence or absence of NTNA.

### Preparation of 4sU hTR substrate

DNA templates for in vitro transcription were generated by PCR. BLoli6336 and BLoli6790 as listed in Supplementary Table 3 were used in a PCR reaction to generate the template for the 5′ hTR fragment. The 3′ RNA fragments that contain a single photoactive 4sU were purchased from GE Dharmacon and are listed in Supplementary Table 10. The 3′ fragment was labeled with ^32^P at the 5′ end using T4 polynucleotide kinase. hTR substrates containing a single ^32^P-labeled 4sU were produced by ligation of a 3′ fragment to the 3′ end of the in vitro-transcribed 5′ fragment using T4 RNA ligase 2 (Rnl2, NEB). A splint DNA oligonucleotide (BLoli6792 as listed in Supplementary Table 10) was mixed with the 5′ and 3′ fragments at a ratio of 2:1:3 (Splint:5′fragment:3′fragment) for ligation in a buffer containing 50 mM Tris-HCl (pH 7.5), 10 mM MgCl_2_, 1 mM DTT, 1 mM ATP, 2 units µl^−1^ RNasin plus RNase inhibitor (Promega), and 1 unit µl^−1^ T4 RNA ligase II. The ligated products were gel-purified by electrophoresis on a 6% acrylamide sequencing gel containing 8 M urea.

### Crosslinking analysis

The 4sU–hTR substrates (6 nM) were incubated at 37 °C for 10 min in buffer containing 20 mM Tris-HCl (pH 7.5), 50 mM KCl, 2.5 mM MgCl_2_, 50 µg ml^−1^ BSA (NEB), and 40 U RNasin plus RNase inhibitor (Promega). The reaction mixture was spread onto a piece of parafilm covering an ice-cold aluminum block. Droplets were UV-irradiated for 20 min at a distance of ~2 cm with a 365-nm UV Lamp (Model UVGL-25, UVP Inc.). The crosslinking products were analyzed on a 6% acrylamide sequencing gel containing 8 M urea.

### Primer extension

The crosslinking products were gel-purified by electrophoresis on a 6% acrylamide sequencing gel containing 8 M urea. The crosslinking sites were mapped by primer extension. Purified RNA was incubated with ^32^P-labeled oligonucleotide BLoli1915 (ACGTCCCACAGCTCAGGGAAT) or BLoli5880 (GCATGTGTGAGCCGAGTCCTGG) and dNTPs (10 nmol) in 6.5 µl of ddH_2_O at 65 °C for 5 min and at 55 °C for 2 min. The reaction volume was increased to 10 µl by the addition of RNase inhibitor (RNasin Plus, 40 U), dithiothreitol (5 mM), 1× first-strand buffer (Invitrogen), and Superscript III reverse transcriptase (200 U, Invitrogen). Reaction mixtures were incubated at 55 °C for 60 min and terminated by the addition of an equal volume of formamide dye. Primer extension products were separated on 12% acrylamide sequencing gel containing 8 M urea next to a sequencing ladder as a marker. Sequencing reactions contained the same primer as used for the primer extension, pMG80 as template, and components of the Sequenase^TM^ Version 2.0 DNA Sequencing Kit (US Biologicals) as instructed by the manufacturer.

### Reporting Summary

Further information on experimental design is available in the Nature Research Reporting Summary linked to this article.

## Supplementary information

Supplementary Information

Source Data

## Data Availability

Custom Python scripts used as part of this study are available at https://github.com/baumannlab/hTR-3prime-RACE. The primary sequence data associated with this analysis has been deposited under GEO accession number GSE111081. Original data underlying gels and blots in this manuscript can be accessed from the Stowers Original Data Repository at http://www.stowers.org/research/publications/LIBPB-1296. Uncropped scans of blots shown in Figs. 2a, c, e; 3a, b; 4a, c; and 5d and f are also included in the Supplementary data associated with this manuscript. A source data file has been provided. A reporting summary for this Article is available as a Supplementary Information file. All reagents described in this study are available upon reasonable request.
