# Supplementary material for: The H/ACA complex disrupts triplex in hTR precursor to permit processing by RRP6 and PARN
Source: Nat Commun. 2018 Dec 21;9:5430. doi: 10.1038/s41467-018-07822-6 (PMC6303318; doi:10.1038/s41467-018-07822-6)
Supplement: Supplementary file 1 — Supplementary Information [file 41467_2018_7822_MOESM1_ESM.pdf]

Supplementary information

The H/ACA complex disrupts triplex in hTR precursor to permit processing by RRP6 and PARN

Tseng et al.

Supplemental Tables:

Supplemental Table 1. Long RNA oligos corresponding to hTR sequence used in this study:

Name	Sequence
451	GGGACGUGCACCCAGGACUCGGCUCACACAUGC
451+5a	GGGACGUGCACCCAGGACUCGGCUCACACAUGCaaaa
453	GGGACGUGCACCCAGGACUCGGCUCACACAUGCAG
453+5a	GGGACGUGCACCCAGGACUCGGCUCACACAUGCAGaaaa
455	GGGACGUGCACCCAGGACUCGGCUCACACAUGCAGUU
455+5a	GGGACGUGCACCCAGGACUCGGCUCACACAUGCAGUUaaaa
460	GGGACGUGCACCCAGGACUCGGCUCACACAUGCAGUUCGCUU
460+5a	GGGACGUGCACCCAGGACUCGGCUCACACAUGCAGUUCGCUUaaaa
455 (452/453 GC)	GGGACGUGCACCCAGGACUCGGCUCACACAUGCGCUU
455+5a (452/453 GC)	GGGACGUGCACCCAGGACUCGGCUCACACAUGCGCUUaaaa

Lower case "a" denotes non-templated adenosines present on a subset of hTR transcripts.

Supplemental Table 2. Short hTR RNA oligos used in this study:

Name	Sequence
S455	GGGACGUGCACCCAGGACUCGGCUCACACAUGCAGUU
S455+5a	GGGACGUGCACCCAGGACUCGGCUCACACAUGCAGUUaaaa
S460	GGGACGUGCACCCAGGACUCGGCUCACACAUGCAGUUCGCUU
S460+5a	GGGACGUGCACCCAGGACUCGGCUCACACAUGCAGUUCGCUUaaaa

Supplemental Table 3. Primers used to generate DNA templates for *in vitro* transcription.

Name	Sequence (T7 promoter sequence is underlined)		Description
BLoli5908	FW	TAATACGACTCACTATAGGGGGGTTGCGGAGGGTGGGCCTGG	5' end at position +1 of hTR
BLoli6336	FW	TAATACGACTCACTATAGGGGACCTGCGGCGGGTCGC	5' end at position +206
BLoli6790	RV	GTGAGCCGAGTCCTGGGTGC	3' end at position +445, used for 4sU RNA preparation
BLoli5880	RV	GCATGTGTGAGCCGAGTCCTGG	3' end at position +451
BLoli5982	RV	TTTTTGCATGTGTGAGCCGAGTCCTGG	3' end at position +451+5a
BLoli5987	RV	AACTGCATGTGTGAGCCGAGTCCT	3' end at position +455
BLoli5988	RV	TTTTTAACTGCATGTGTGAGCCGAGTCCT	3' end at position +455+5a
BLoli6914	RV	AGCGAACTGCATGTGTGAGCCGA	3' end at position +459
BLoli6915	RV	TTTTTAGCGAACTGCATGTGTGAGCCGA	3' end at position +459+5a
BLoli5989	RV	AAGCGAACTGCATGTGTGAGCCGA	3' end at position +460
BLoli5990	RV	TTTTTAAGCGAACTGCATGTGTGAGCCGA	3' end at position +460+5a
BLoli6758	RV	AAAGCGAACTGCATGTGTGAGCCGA	3' end at position +461
BLoli6759	RV	TTTTTAAAGCGAACTGCATGTGTGAGCCGA	3' end at position +461+5a
BLoli6996	RV	AGAGCGAACTGCATGTGTGAGCCGA	3' end at position +461 (U460C)
BLoli6997	RV	TTTTTAGAGCGAACTGCATGTGTGAGCCGA	3' end at position +461+5a (U460C)

Supplemental Table 4. Oligos used for site-directed mutagenesis

Name	Sequence		Description
BLoli6973	FW	CACATGCAGTTCGCTCTCCTGTTGGTGGGG	T460C
BLoli6974	RV	CCCCACCAACAGGAGAGCGAACTGCATGTG	
BLoli6830	FW	CTTTCAGGCCGCAGGAAGAATAACGGAGCGAGTCCCCGC	U92

BLoli6831	RV	GCGGGGACTCGCTCCGTTATTCTTCCTGCGGCCTGAAAG	

Supplemental Table 5. Plasmids used for transfection.

Plasmid name	description
pMG80	WT hTR in pACGFP1-1
pHF106	T460C hTR in pACGFP1-1
pHF84	U92 hBOX hTR in pACGFP1-1

Supplemental Table 6. siRNAs used in this study

Target	sequences (5' to 3')
siGENOME Non-targeting siRNA Pool #1<br>(controls)<br>(Dharmacon)	Ctrl-1: AUGCGACUAAACACAUCAA<br>Ctrl-2: UAAGGCUAUGAAGAGAUAC<br>Ctrl-3: AUGUAUUGGCCUGUAUUAG<br>Ctrl-4: AUGAACGUGAAUUGCUCAA
RRP6	RRP6-1: CAAAAUCUGAAACUUUCCdTdT<br>RRP6-2: CAGUUAUACAGACCUAUAdTdT
RRP40	CACGCACAGUACUAGGUCAdTdT
PARN	AGGCAUUCAUGUUGAGACUdTdT
DKC1 Stealth siRNA (HSS102781)	DKC1-1: GGCCAAGAUUAUGCUUCCAGGUGUU<br>DKC1-2: AACACCUGGAAGCAUAAUCUUGGCC

Supplemental Table 7. Antibodies used for western blotting.

Target	Source	Cat#/reference	Dilution
RRP6	Proteintech Group	11178-1-AP	1:1000
RRP40	Bethyl Laboratories	A303-909A	1:1000
TUBULIN	SIGMA-ALDRICH	T5168	1:20000
PARN	Bethyl Laboratories	A303-562A	1:4000
DKC1	Santa Cruz Biotechnology	sc-48794	1:2000

Supplemental Table 8. Oligonucleotides used for probe preparation for northern blots.

Name	Sequences		Target
BLoli1309	FW	GGGTTGCGGAGGGTGGGC	hTR probe generated by PCR
BLoli1292B	RV	CCGACTTTGGAGGTGCCTTC	
BLoli2948		AGCAGCCGATTGTCTGTTGTGCCCAGT	Neomycin probe

Supplemental Table 9. Oligonucleotides used for 3' RACE followed by Illumina sequencing.

Name		Sequences	Description
BLoli5511		5rApp/TTTAACCGCGAATTCCAG/3ddC	3' linker
BLoli5575	RV	GACTGGAGTTCAGACGTGTGCTCTTCCGATCT CTGGAATTCGCGGTTAAA	RT primer
BLoli5574	FW	CCTACACGACGCTCTTCCGATCTNNNNNNNNNNCAGG AAGAGGAACGGAGCGAGT	First round PCR of 3' RACE
BLoli5575	RV	GACTGGAGTTCAGACGTGTGCTCTTCCGATCT CTGGAATTCGCGGTTAAA	
BLoli4666	FW	AATGATACGGCGACCACCGAGATCTACACTCTTTCCC TACACGACGCTCTTCCGATC	Second round PCR of 3' RACE
BLoli4668	RV	CAAGCAGAAGACGGCATACGAGATCGTGATGTGACT GGAGTTCAGACGTGTG	
BLoli4669	RV	CAAGCAGAAGACGGCATACGAGATACATCGGTGACT GGAGTTCAGACGTGTG	

BLoli4779	RV	CAAGCAGAAGACGGCATACGAGATGTAGCCGTGACT GGAGTTCAGACGTGTG	
BLoli4780	RV	CAAGCAGAAGACGGCATACGAGATTACAAGGTGACT GGAGTTCAGACGTGTG	

Supplemental Table 10. RNA oligonucleotides used in generating 4sU labelled hTR fragments:

Name	Sequence	4sU position
BLoli6923	ACAUGCAGUUCGC(4-S-U)UU	459 (WT)
BLoli6924	ACAUGCAGUUCGCU(4-S-U)U	460 (WT)
BLoli6922	ACAUGCAGUUCGCUU(4-S-U)A	461 (WT)
BLoli6987	ACAUGCAGUUCGCUC(4-S-U)A	461 (U460C)
BLoli6792:	AAAGCGAACTGCATGTGTGAGCCGAGTCCTGGGTGC	DNA splint

Supplementary Figure 1.

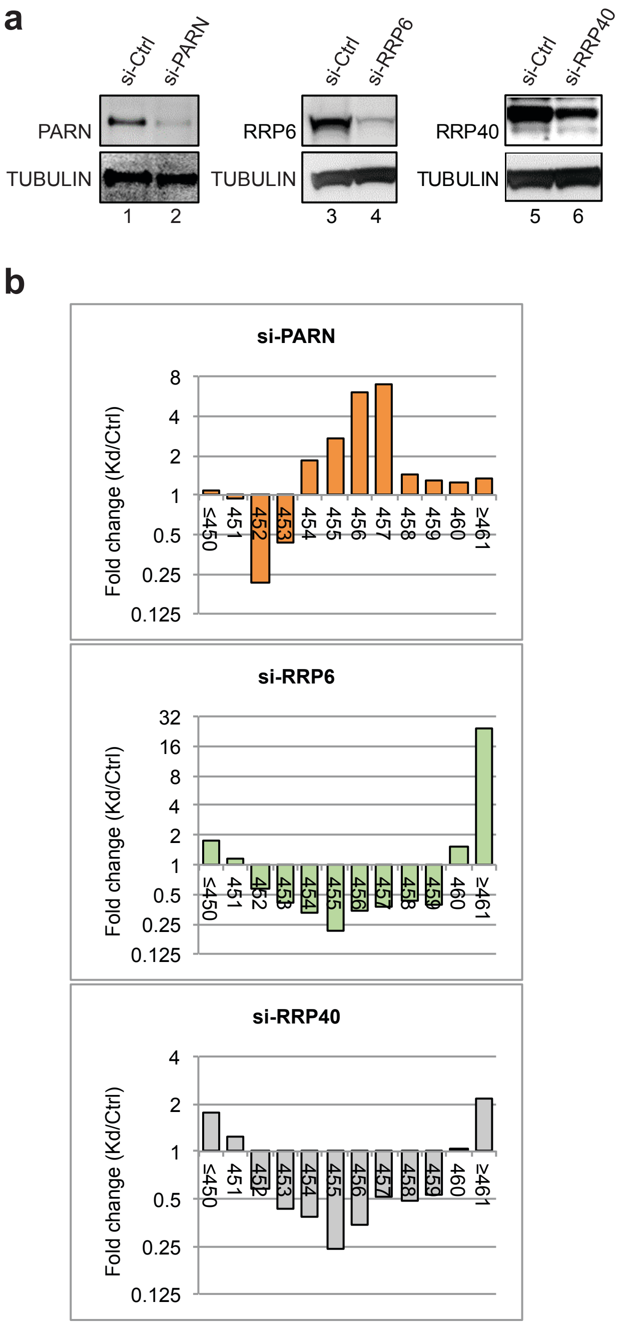

Supplementary Figure 1. (**a**) Western blotting analysis of cell extracts prepared from HeLa cells treated with siRNAs targeting PARN, RRP6, and RRP40. Non-targeting siGENOME pool was used as control. Endogenous TUBULIN served as a loading control. (**b**) Graphical representation of changes in distribution of hTR 3' end positions.

Supplementary Figure 2.

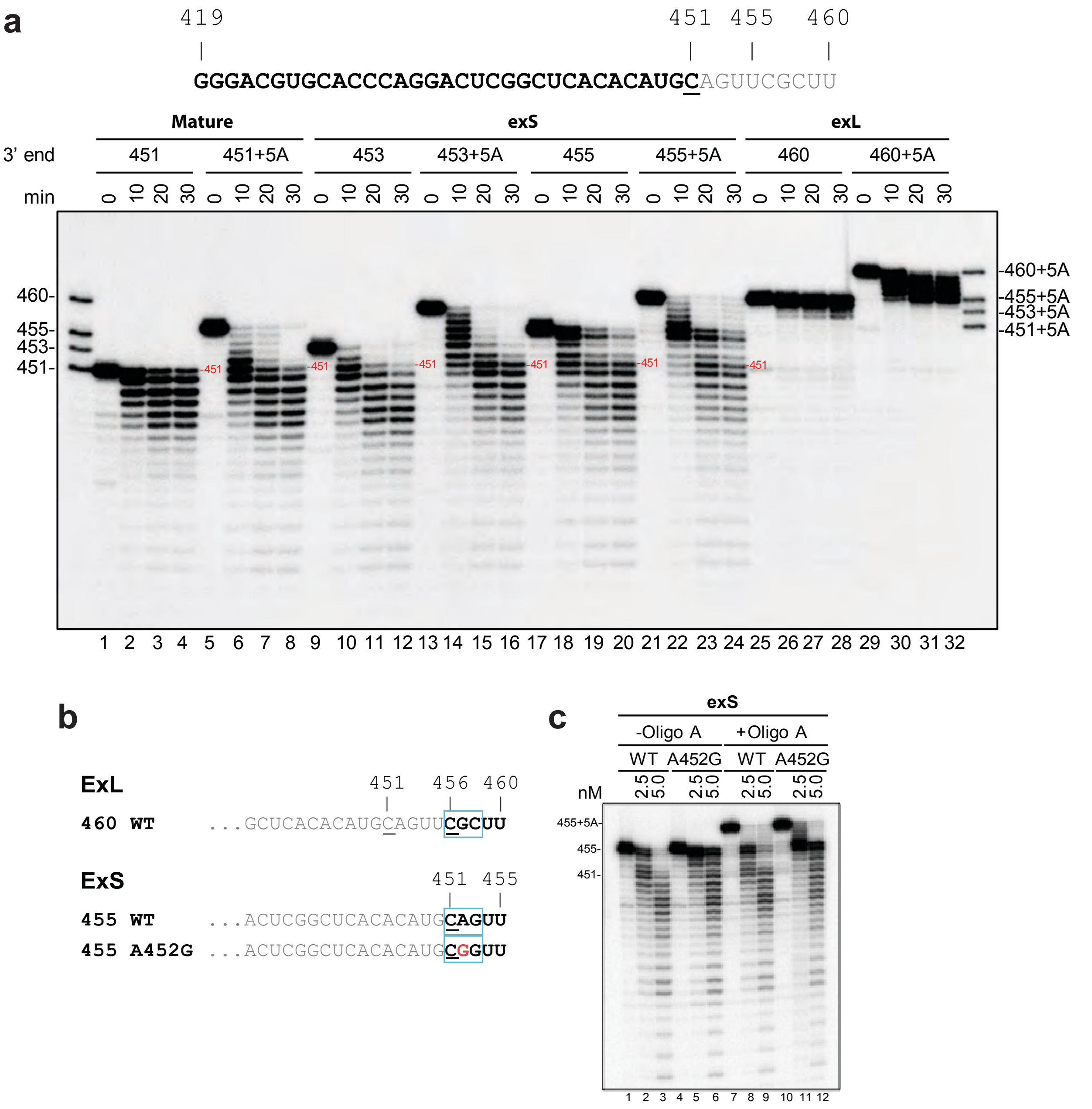

Supplementary Figure 2. (**a**) 5'-^{32}P-labeled hTR sequences (nucleotide 419 to different 3' ends as the indicated with or without oligo(A) tails) were incubated without or with 2.5 nM of recombinant PARN at 37°C for the indicated times. The reaction products were resolved on a 20% polyacrylamide gel under denaturing conditions. (**b**) Schematic of hTR oligos used in Supplementary Fig. 2c. (**c**) 5'-^{32}P-labeled hTR sequences were incubated without or with 2.5, or 5.0 nM of recombinant PARN at 37°C for 30 min. The reaction products were resolved on a 20% polyacrylamide gel under denaturing conditions.

Supplementary Figure 3

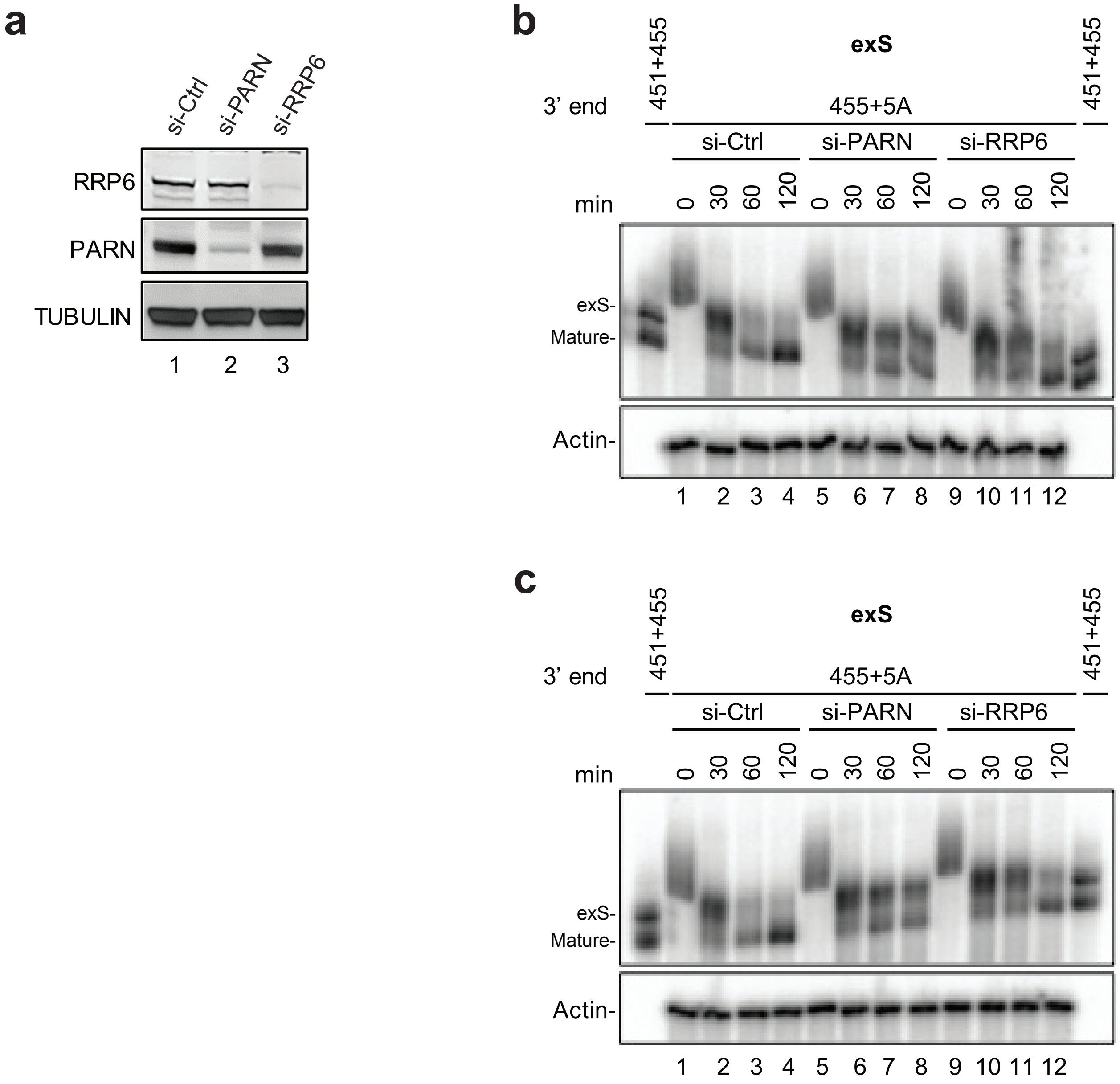

Supplementary Figure 3. (a) Western blotting analysis of cell extracts prepared from HeLa cells treated with siRNAs targeting PARN or RRP6. The nontargeting siGENOME pool was used as control. Endogenous TUBULIN served as a loading control. **(b and c)** In vitro hTR processing assays (two independent experiments). The 32P-labeled in vitro transcribed hTR fragments (nucleotide 206 to 455 with oligo A tails) was incubated in cell extracts (20 µg) prepared from siRNA treated cells as indicated. The reaction was performed at 37oC for the indicated times. RNA was purified and resolved by a 6% polyacrylamide /8M urea gel.

Supplementary Figure 4.

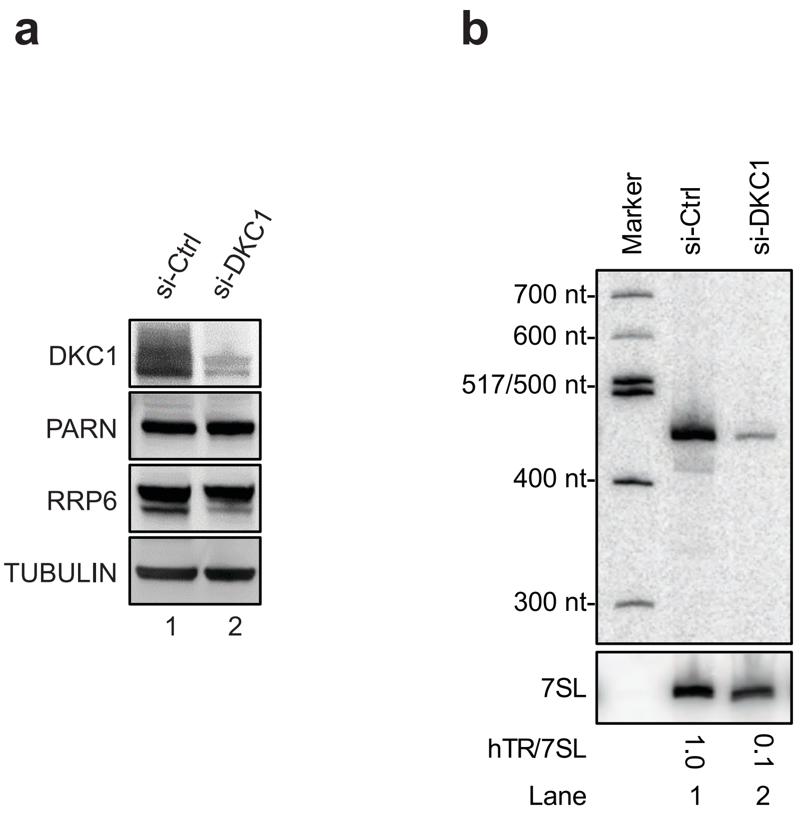

Supplementary Figure 4. (**a**) Western blotting analysis of cell extracts prepared from HeLa cells treated with siRNAs targeting DKC1. Nontargeting siGENOME pool was used as control. Endogenous TUBULIN served as a loading control. (**b**) Northern blotting analysis of hTR from HeLa cells treated with siRNA against dyskerin. Nontargeting siGENOME pool was used as control. A probe against 7SL RNA served as a loading control.

Supplementary Figure 5.

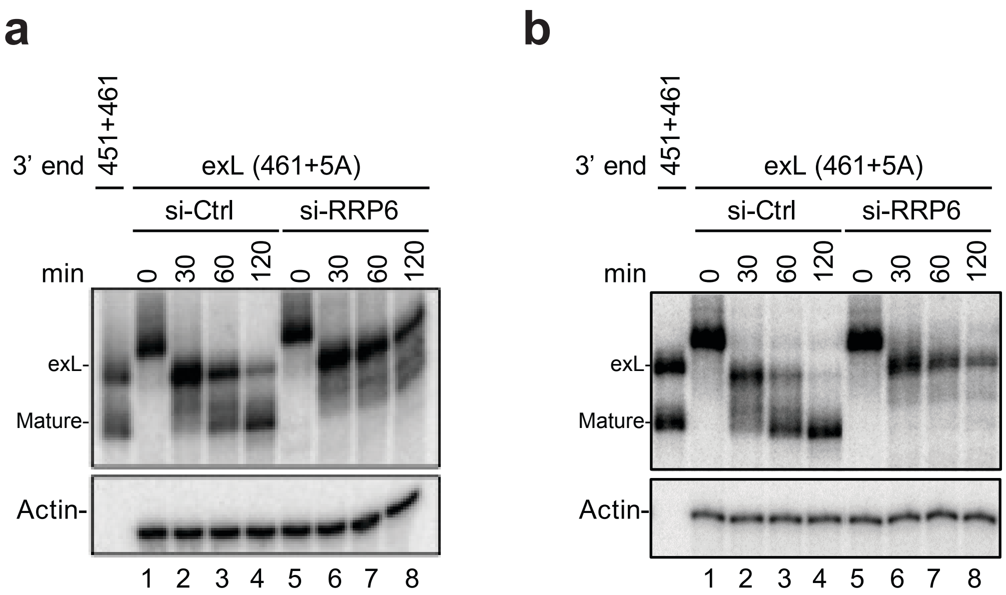

Supplementary Figure 5. (**a** and **b**) *In vitro* hTR processing assays from two independent experiments. The ^{32}P-labeled *in vitro* transcribed hTR fragments (nucleotide 206 to 461 with oligo A tails) was incubated in cell extracts (20 µg) prepared from RRP6 siRNA treated cells. The reaction was performed at 37°C for the indicated times. RNA was purified and resolved by a 6% polyacrylamide/8M urea gel.

Supplementary Figure 6.

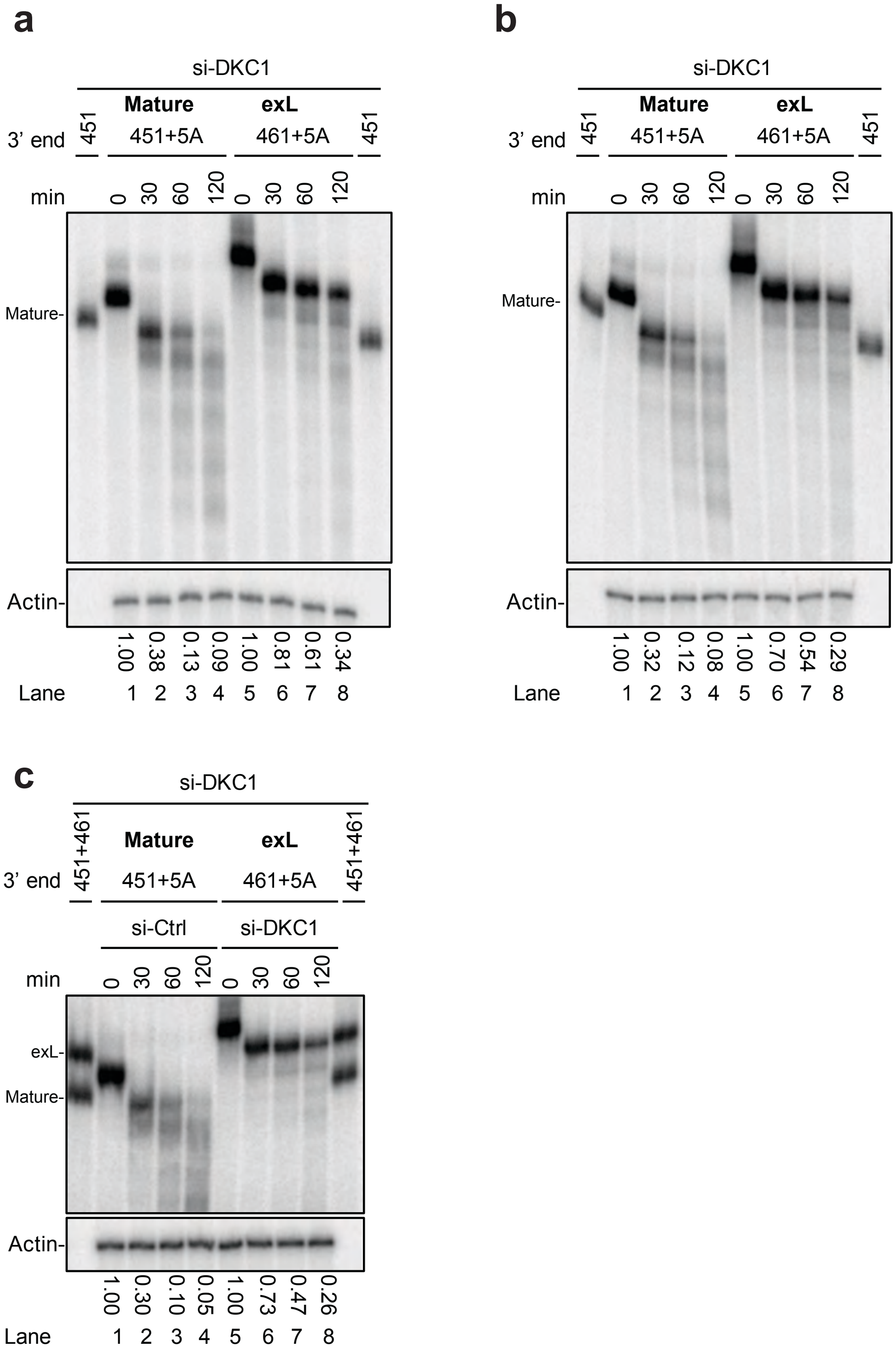

Supplementary Figure 6. (**a-c**) *In vitro* hTR processing assays from three independent experiments. The ^{32}P-labeled *in vitro* transcribed hTR fragments (nucleotide 206 to 451 and 461 with oligo A tails) were incubated in dyskerin knockdown extracts at 37°C for the indicated times. RNA was purified and resolved on a 6% polyacrylamide/8M urea gel.

Supplementary Figure 7.

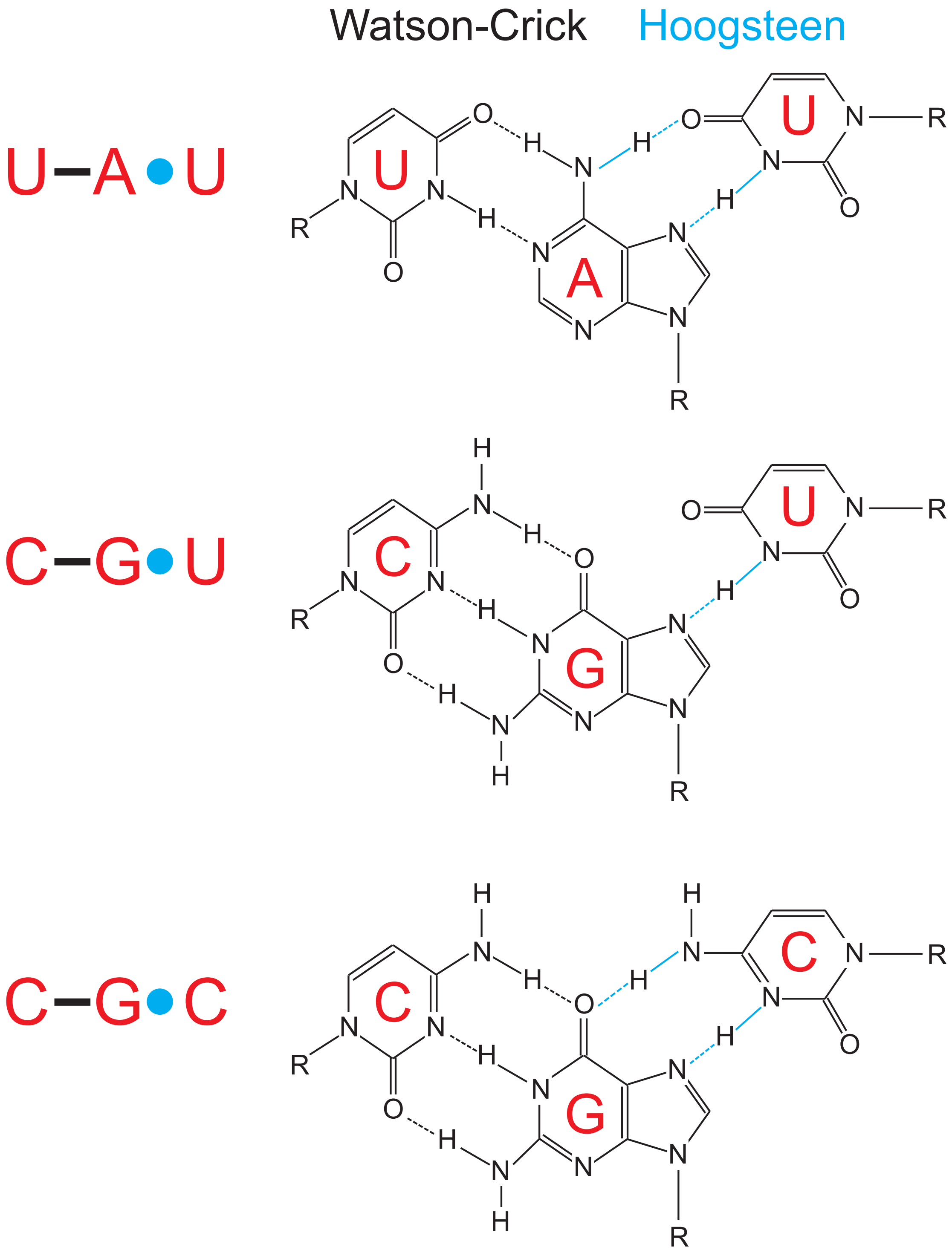

Supplementary Figure 7. Base triples formed by U-A●U, C-G●C and C-G●U, respectively. - represents a Watson-Crick interaction as shown in black and ● represents a Hoogsteen interaction as shown in blue.

Supplementary Figure 8.

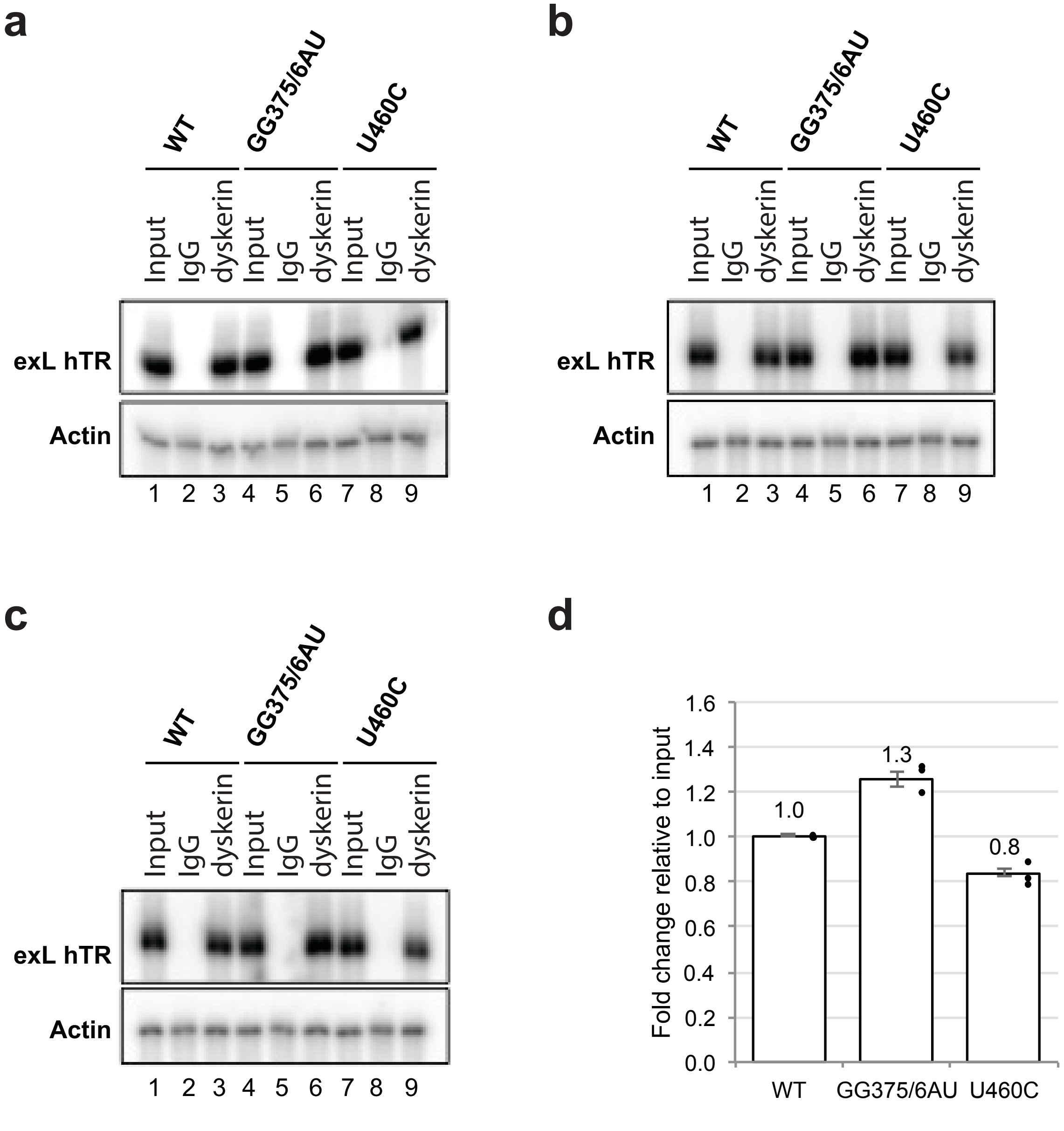

Supplementary Figure 8. (a-c) *In vitro* immunoprecipitation assays from three independent experiments. Wild type or mutant hTR fragments (nucleotide 206 to 461 with or without oligo A tails) as indicated were *in vitro* transcribed in the presence of α-^{32}P-UTP and incubated in HeLa cell extracts at 37°C for 30 min. The reaction mixture was immunoprecipitated with antibodies against dyskerin. RNA was purified and resolved by a 6% polyacrylamide/8M urea gel. (**d**) The bar graph illustrates the mean fold change for hTR levels relative to input samples and normalized to actin. Mean values were calculated from three independent experiments of three biological replicates. Bars represent the standard error. Significance of change in hTR level between samples was calculated with a two-sided Student's t-test; P values: 0.001873 (GG375/6AU); 0.000844 (U460C). Dots represent data points from individual experiments.

Supplementary Figure 9.

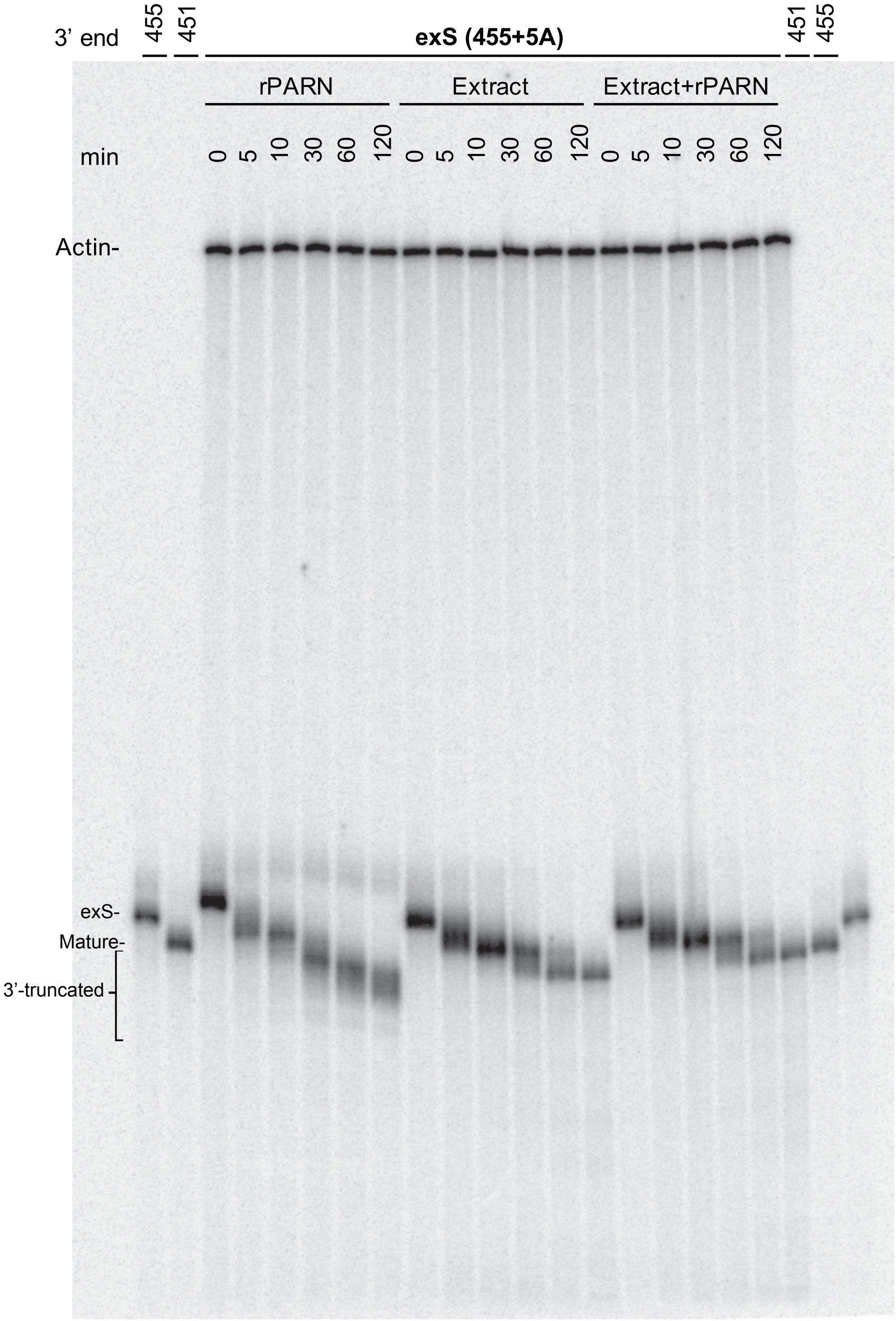

Supplementary Figure 9. Full blot of Figure 2b.

Supplementary Figure 10.

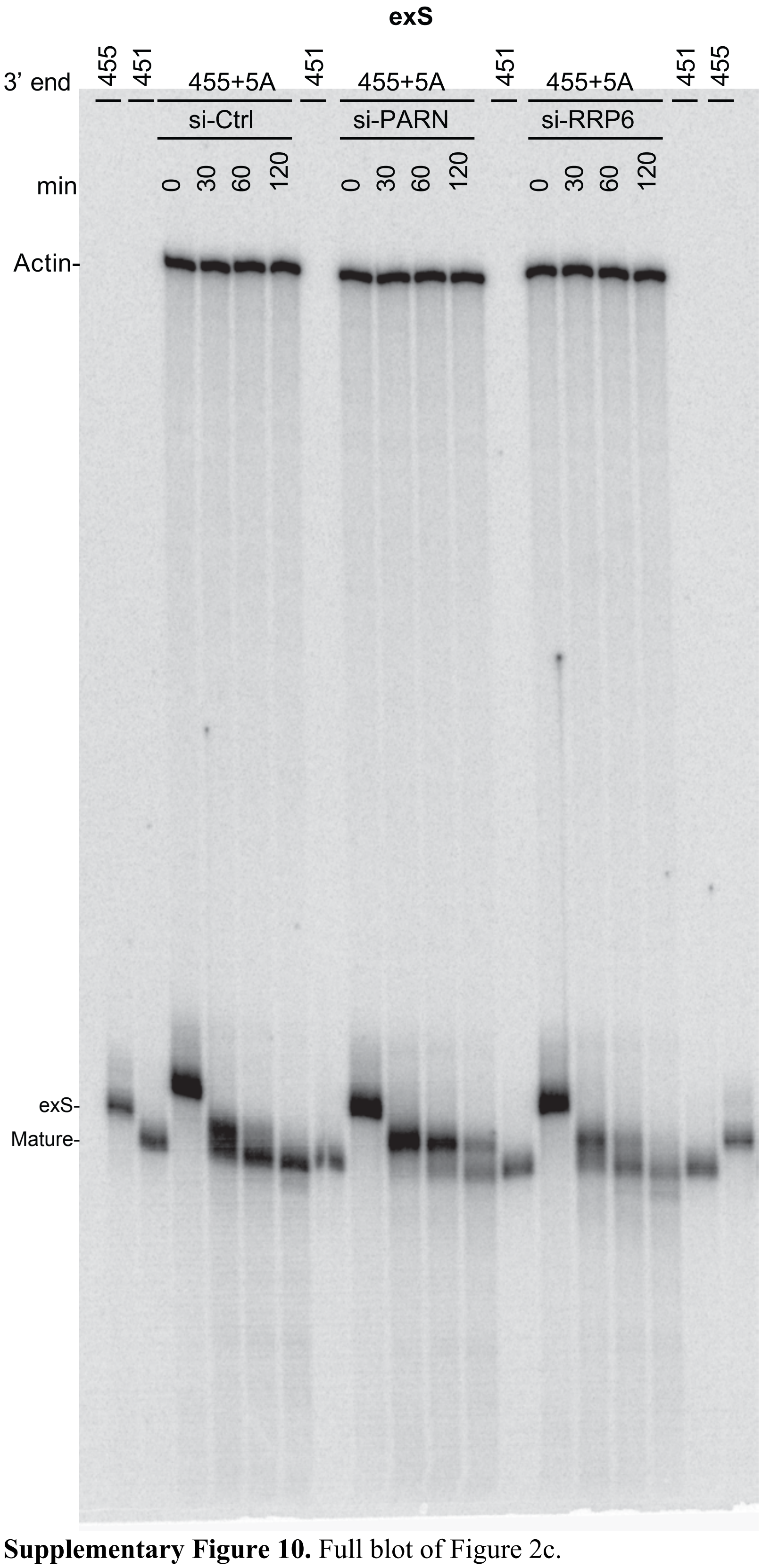

Supplementary Figure 10. Full blot of Figure 2c.

Supplementary Figure 11.

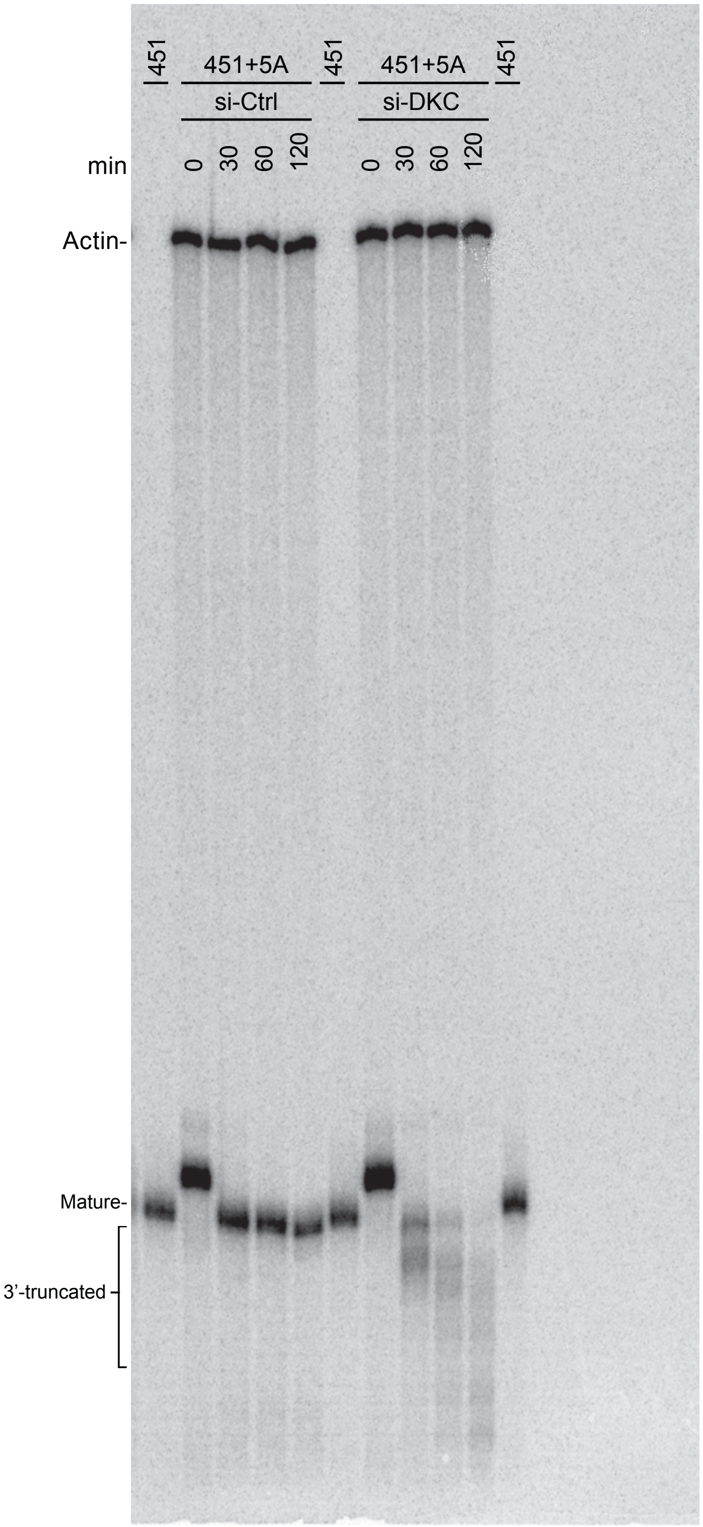

Supplementary Figure 11. Full blot of Figure 2e.

Supplementary Figure 12.

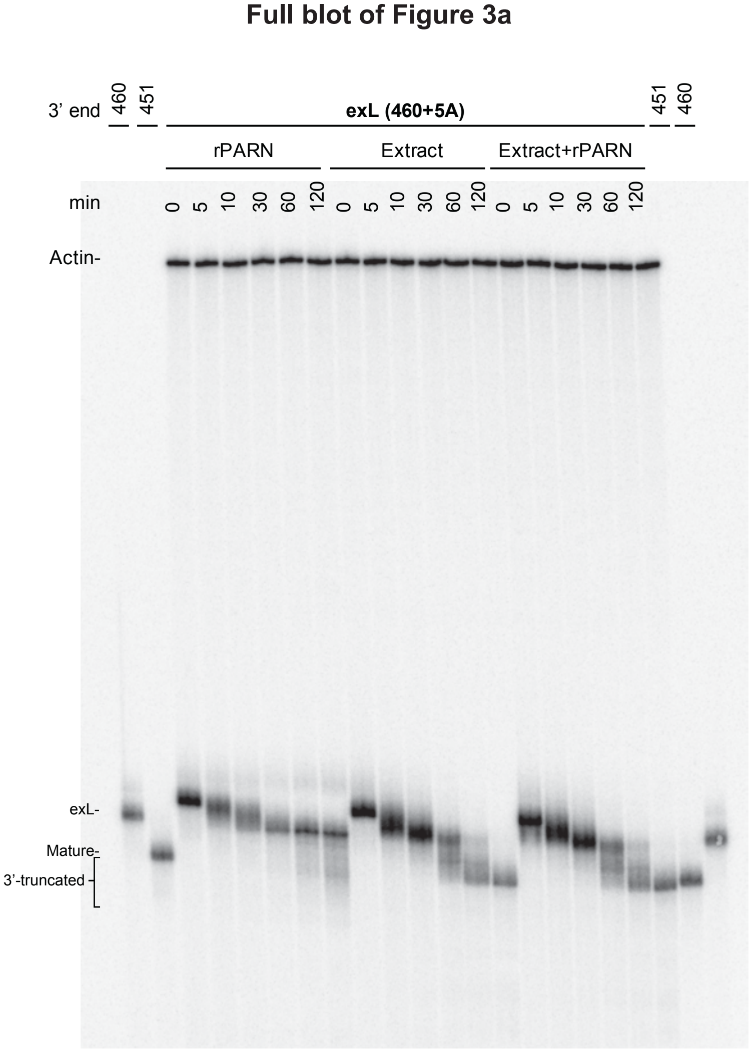

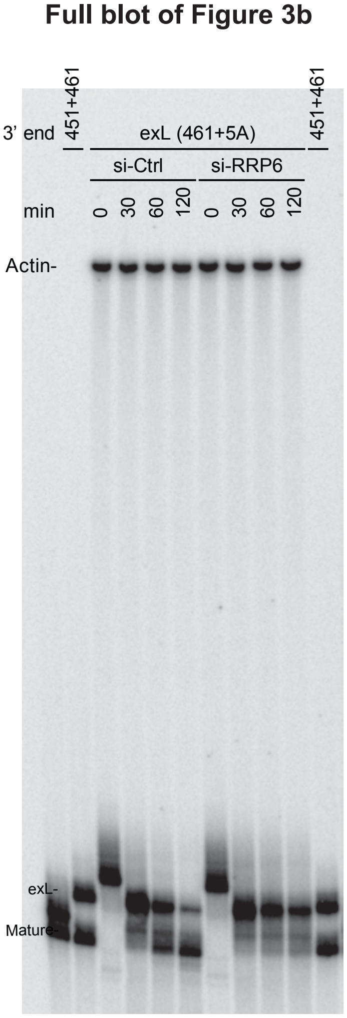

Supplementary Figure 12. Full blots of Figure 3a and 3b.

Supplementary Figure 13.

Full blot of Figure 4a

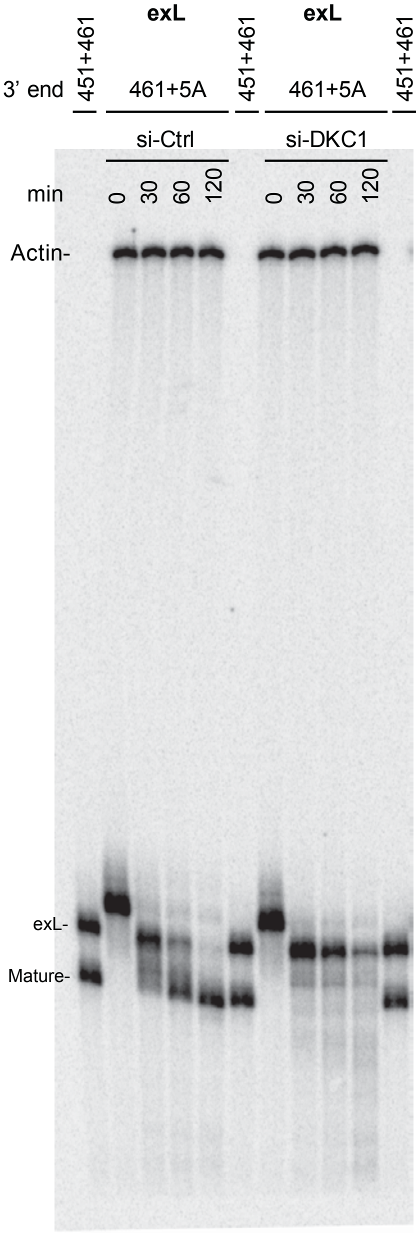

Full blot of Figure 4c

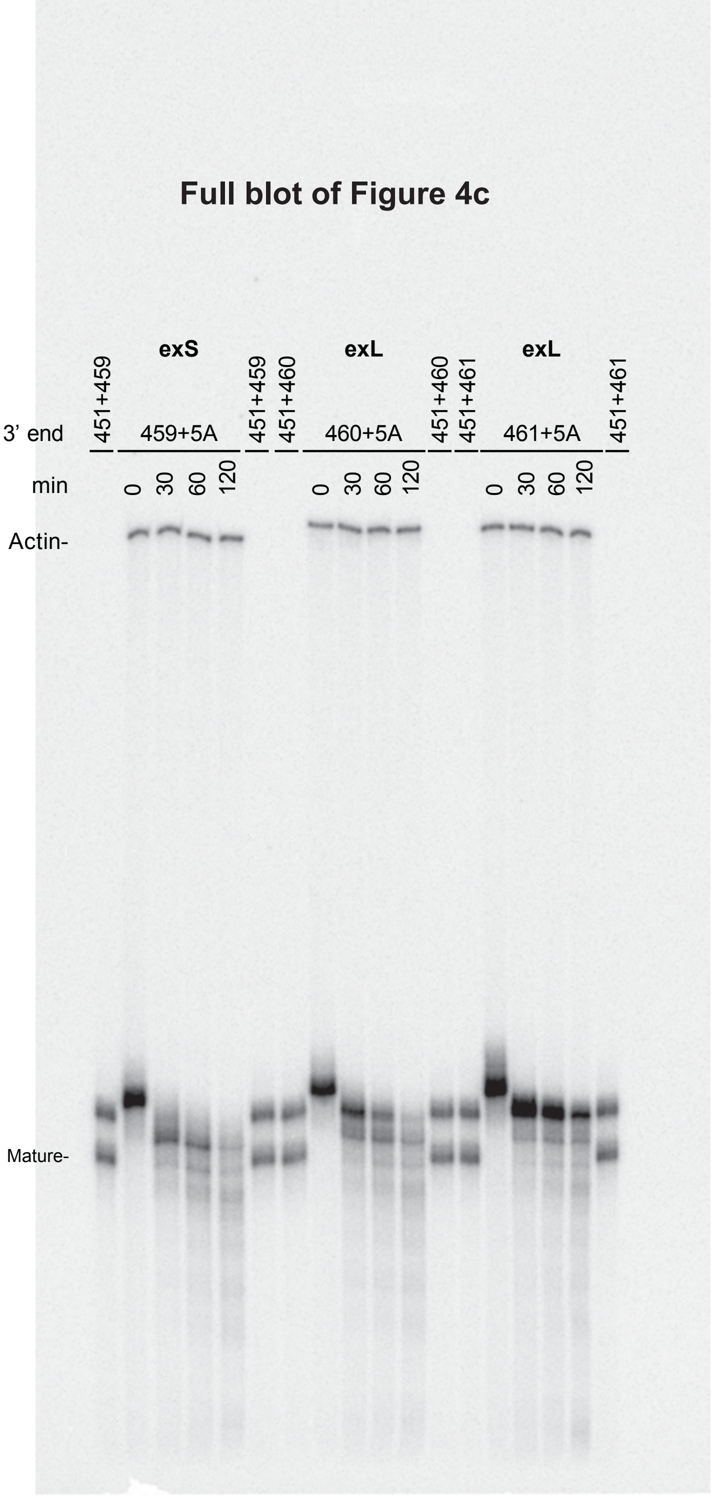

Supplementary Figure 13. Full blots of Figure 4a and 4c.

Supplementary Figure 14.

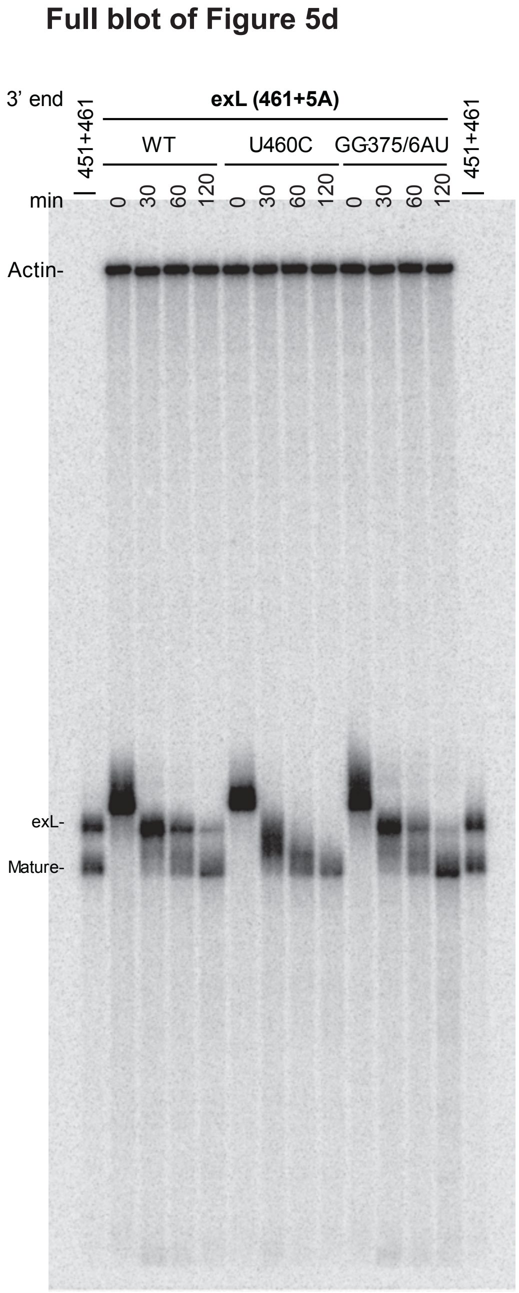

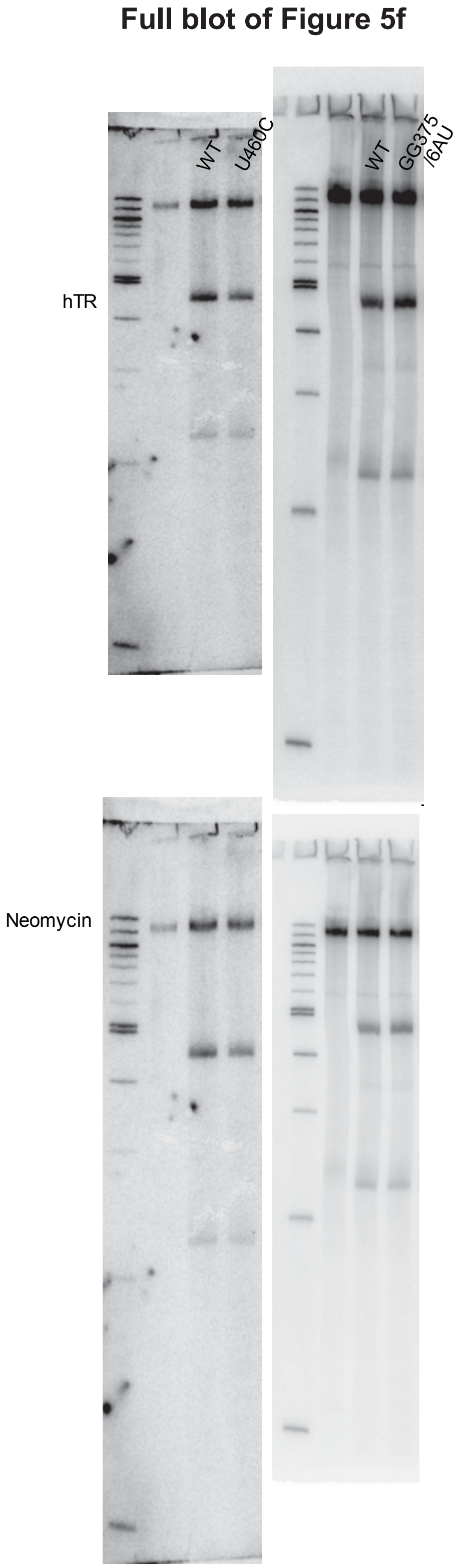

Supplementary Figure 14. Full blots of Figure 5d and 5f
